# Antigen reactivity defines tissue-resident memory and exhausted T cells in tumors

**DOI:** 10.1038/s41590-025-02347-9

**Published:** 2025-12-29

**Authors:** Thomas N. Burn, Jan Schröder, Luke C. Gandolfo, Maleika Osman, Elanor N. Wainwright, Enid Y. N. Lam, Keely M. McDonald, Rachel B. Evans, Shihan Li, Daniel Rawlinson, Lachlan Dryburgh, Ali Zaid, Zoltan Maliga, Dominick Schienstock, Philippa Meiser, Hyun Jae Lee, Hongjin Lai, Marcela L. Moreira, Pirooz Zareie, Louis H-Y. Lee, Lutfi Huq, Susan N. Christo, Justine J. W. Seow, Keith A. Ching, Stéphane M. Guillaume, Kathy Knezevic, Simone L. Park, Maximilien Evrard, Jason Waithman, Thomas Gebhardt, Scott N. Mueller, Georgina E. Riddiough, Marcos V. Perini, Simon C. H. Tsao, Terence P. Speed, Peter K. Sorger, Sherene Loi, Francis R. Carbone, Stephanie Gras, Timothy S. Fisher, Bas J. Baaten, Mark A. Dawson, Laura K. Mackay

**Affiliations:** 1 Department of Microbiology and Immunology, The University of Melbourne at the Peter Doherty Institute for Infection and Immunity, Melbourne, Victoria Australia; 2 Department of Microbiology and Immunology, Computational Science Initiative, The University of Melbourne at the Peter Doherty Institute for Infection and Immunity, Melbourne, Victoria Australia; 3 Bioinformatics Division, Walter and Eliza Hall Institute of Medical Research, Melbourne, Victoria Australia; 4 School of Mathematics and Statistics, University of Melbourne, Melbourne, Victoria Australia; 5 Peter MacCallum Cancer Centre, Melbourne, Victoria Australia; 6 The Sir Peter MacCallum Department of Medical Oncology, University of Melbourne, Melbourne, Victoria Australia; 7 Laboratory of Systems Pharmacology, Harvard Medical School, Boston, MA USA; 8 Department of Thoracic Surgery and Institute of Thoracic Oncology, West China Hospital, Sichuan University, Chengdu, China; 9 Oncology Research Unit, Pfizer Inc, San Diego, CA USA; 10 School of Biomedical Sciences, The University of Western Australia, Perth, Western Australia Australia; 11 Telethon Kids Institute, The University of Western Australia, Perth, Western Australia Australia; 12 Department of Surgery, The University of Melbourne, Heidelberg, Victoria Australia; 13 HPB and Liver Transplant Unit, Austin Health, Heidelberg, Victoria Australia; 14 School of Natural Sciences, Macquarie University, Sydney, New South Wales Australia; 15 Infection and Immunity Program, La Trobe Institute for Molecular Science (LIMS), Bundoora, Victoria Australia; 16 Department of Biochemistry and Chemistry, La Trobe University, Bundoora, Victoria Australia; 17 Department of Biochemistry and Molecular Biology, Monash University, Clayton, Victoria Australia; 18 Present Address: Institute for Immunology and Immune Health, Perelman School of Medicine, University of Pennsylvania, Philadelphia, PA USA

**Keywords:** Immunological memory, Immunotherapy

## Abstract

CD8^+^ T cells are an important weapon in the therapeutic armamentarium against cancer. While CD8^+^CD103^+^ T cells with a tissue-resident memory T (T_RM_) cell phenotype are associated with favorable prognoses, the tumor microenvironment also contains dysfunctional exhausted T (T_EX_) cells that exhibit a variety of T_RM_-like features. Here we deconvolute T_RM_ and T_EX_ cells across human cancers, ascribing markers and gene signatures that distinguish these populations and enable their functional distinction. Although T_RM_ cells have superior functionality and are associated with long-term survival post-tumor resection, they are not associated with responsiveness to immune checkpoint blockade. Tumor-associated T_EX_ and T_RM_ cells are clonally distinct, with the latter comprising tumor-independent bystanders and tumor-specific cells segregated from cognate antigen. Intratumoral T_RM_ cells can be forced toward an exhausted fate when chronic antigen stimulation occurs, indicating that the presence or absence of continuous antigen exposure within the microenvironment is the key distinction between tumor-associated T_EX_ and T_RM_ populations. These results highlight unique functions for T_RM_ and T_EX_ cells in tumor control, underscoring the need for distinct strategies to harness these populations for cancer therapies.

## Abstract (web summary)

Here the authors show that tissue-resident memory and exhausted T cells in tumors are distinct populations that are shaped by relative presence or absence of TCR signals, suggesting that a tailored therapeutic strategy is needed to target each subset.

## Main

T cell-mediated tumor control is a key facet of cancer immunotherapy, and pinpointing the most effective T cell subtypes for therapeutic targeting is a critical area of research. Two subsets of substantial interest are T_RM_ cells and T_EX_ cells. CD8^+^ T_RM_ cells are a nonrecirculating memory T cell population that reside in every organ examined^1–3^. Canonical T_RM_ cells develop following the resolution of infection or inflammation, where they can provide rapid, localized immune protection^4,5^. In contrast, CD8^+^ T_EX_ cells form in the context of chronic infection and cancer, driven by persistent antigen recognition and inflammation^6–8^. T cell exhaustion entails loss of proliferation and function and, although immune checkpoint blockade (ICB) therapies temporarily reinvigorate T_EX_ cell function, their long-term fate remains largely unaltered^9^. T_EX_-phenotype cells also become resident in tumors, probably sharing some aspects of the T_RM_ transcriptional program to limit recirculation^10^. Because many studies use genetic signatures to identify T cell subtypes, their transcriptional similarities have resulted in T_RM_ and T_EX_ cells being conflated in the literature^11–16^. Although tumor-associated T_RM_ cells may exist as a potential antipode to the dysfunctional T_EX_ population, their identification within tumors and contribution to cancer control has not been adequately addressed.

## Results

### T_EX_ cells share the T_RM_ gene signature

**Fig. 1:**
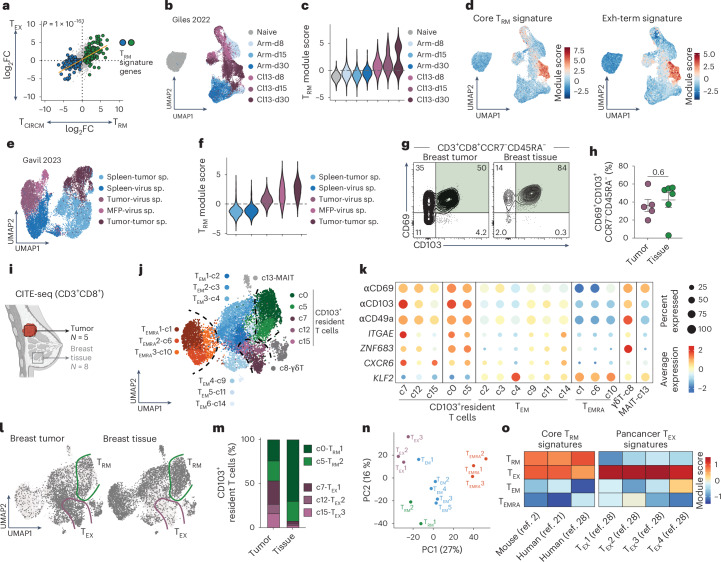
Canonical T_RM_ proteins and gene signatures do not deconvolute T_RM_ and T_EX_ cells. **a**, The log_2_FC of differentially expressed T_RM_ (ref. ^2^) and T_EX_ (ref. ^26^) cell genes compared to T_CIRCM_. T_RM_ signature genes^2^ are highlighted (green, up; blue, down); *P* value indicates two-sided Fisher’s exact test for association. **b**–**d**, scRNA-seq data from LCMV-specific T cells after acute (Arm) or chronic (Cl13) infection isolated from spleens at day 8 (d8), day 15 (d15) or day 30 (d30) postinfection^27^: UMAP projection of scRNA-seq data annotated by infection and timepoint (**b**), quantification of T_RM_ module score^2^ (**c**) and overlay of core T_RM_ (ref. ^2^) and terminally exhausted (Exh-Term)^27^ transcriptional signatures on respective populations (**d**). **e**,**f**, UMAP projection (**e**) and quantification of T_RM_ module score^2^ (**f**) on LCMV-specific P14 (virus-sp.) or tumor-specific (tumor-sp.) OT-I T cells from CITE-seq analysis from EO771-OVA BC tumors, surrounding mammary fat pad (MFP) and spleen^10^. **g**,**h**, Flow cytometry of CD8^+^ T cells isolated from BC tumors or breast tissue from *N* = 5 BC patients. Representative plots (**g**) and summary data for percentage of CD69^+^CD103^+^CCR7^−^CD45RA^−^ of CD8^+^ T cells (**h**). Error bars, mean ± s.e.m. **i**–**o**, CITE-seq of CD3^+^CD8^+^ T cells from primary BC tumors (*N* = 5) and noncancerous breast tissue (*N* = 8): **i**, Schematic. **j**, Data were Harmony integrated, and unified protein and RNA-seq data were represented on weighted nearest neighbors UMAP and colored by cluster. **k**, Expression of respective cell-surface proteins (αCD103, αCD69, αCD49a) and transcripts (*ITGAE*, *ZNF683*, *CXCR6*, *KLF2*) across annotated clusters. **l**–**m**, CD8^+^ T cells segregated by tissue of origin (**l**) and relative cluster composition of CD103^+^ resident T cells isolated from BC tumors or tissue (**m**). **n**, PCA of pseudobulked clusters annotated in **j**. **o**, Average module scores of published T_RM_ (refs. ^2,21,28^) and T_EX_ (ref. ^28^) gene signatures by annotated subsets. Data in **c** and **f** were analyzed by two-sided Wilcoxon rank-sum test with continuity correction, all pairwise comparisons *P* < 1 × 10^−15^. Data in **h** were analyzed by two-sided *t*-test. Illustration in **i** created using BioRender.com. Source data

**Extended Data Fig. 1:**
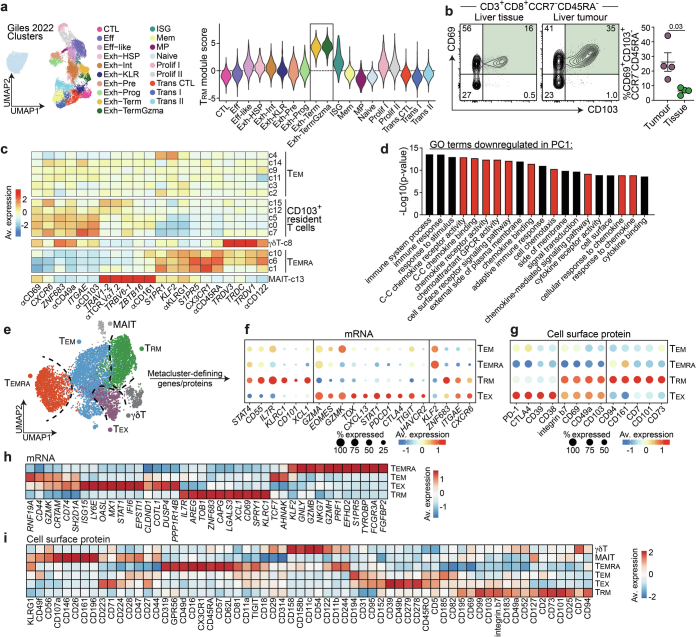
Identification of CD8+ T cell populations in tumours. **a**, T_RM_ gene signature expression on LCMV-specific CD8^+^ T cell clusters from scRNA-seq of the respective dataset^27^ (Related to Fig. 1b–d). **b**, Flow cytometry of CD8^+^ T cells isolated from liver tumours and liver tissue from N=4 colorectal cancer patients with liver metastases. Representative plots and summary data for CD69^+^CD103^+^CCR7^−^CD45RA^−^ CD8^+^ T cells, analysed by two-sided Mann-Whitney test. Error bars indicate mean +/- SEM. **c**, Expression of respective lineage-defining genes and cell surface proteins from BC-CITEseq data (Fig. 1i, j) displayed on heatmap by cluster. **d**, pathway enrichment of downregulated loading genes associated with PC1 from PCA plot in Fig. 1n. Red bars indicate pathways related to chemokine receptor signaling and trafficking **e**, Schematic showing metacluster annotations. **f-g**, Expression of selected shared and distinct T_RM_ and T_EX_ genes (**f**) or cell surface proteins (**g**) across metaclusters from BC dataset. **h-i**, heatmaps showing top DE genes (**h**) and cell surface proteins (**i**) from BC dataset.

Experimental systems such as parabiosis or transplantation have demonstrated the noncirculating behavior of T_RM_ cells^4,5,17^. Such experiments in mice led to the identification of T_RM_-associated markers including CD69 and CD103 that distinguish T_RM_ cells from circulating memory T cells (T_CIRCM_), with markers partially cross-validated in humans within transplanted organs^18–20^. T_RM_ cells defined by CD69 or CD103 expression are transcriptionally distinct from T_CIRCM_ in humans^1,21,22^ and mice^2,23^. At the core of the T_RM_ gene signature exists a transcriptional program designed to halt T cell migration, including downregulation of key regulators of recirculation such as *KLF2*, *S1PR1* and *S1PR5* (refs. ^24,25^). However, T_EX_ cells also cease migration and become resident in tumors^10^. Thus, we hypothesized that T_EX_ cells use a common transcriptional program to T_RM_ cells to inhibit migration, resulting in considerable transcriptional overlap between these populations. Indeed, the T_RM_ transcriptional profile derived from acute viral infection models (lymphocytic choriomeningitis virus (LCMV) and herpes simplex virus (HSV))^2^ correlated significantly with the T_EX_ transcriptional profile derived from chronic versus acute LCMV infection^26^ (Fig. 1a). Given this overlap between T_RM_ and T_EX_ cells, we reasoned that T_RM_ gene signatures would identify T_EX_ cells in single-cell RNA sequencing (scRNA-seq) datasets. To test this, we used published data of CD8^+^ T cells from spleens of mice infected with acute or chronic LCMV^27^. We found the T_RM_ gene signature was most enriched in terminal T_EX_ cells during chronic infection (Fig. 1b–d and Extended Data Fig. 1a). In CD8^+^ T cells from murine breast cancer (BC) and adjacent tissue^10^, the T_RM_ gene signature was highly enriched in tumor-specific T_EX_ cells isolated from tumors, similar to virus-specific cells in adjacent tissue (Fig. 1e,f). Thus, the utilization of T_RM_ gene signatures to identify intratumoral T_RM_ cells results in aberrant T_EX_ cell identification.

Many studies have identified T_RM_ cells in tumors via CD69 and CD103 coexpression^11–16^. CD69^+^CD103^+^ CD8^+^ T cells are present in both noncancerous tissue and tumors, the latter of which probably encompasses a mixed population of T_RM_ and T_EX_ cells (Fig. 1g,h and Extended Data Fig. 1b). Thus, CD69, CD103 and T_RM_ gene signatures all appear insufficient to distinguish T_RM_ and T_EX_ cells should they coexist. Given this overlap in tissue-residency features, we set out to differentiate T_RM_ and T_EX_ populations in human tumors by formulating two testable assumptions. First, that cells enriched for core-residency genes and signatures in healthy, noninflamed tissues are predominantly bona fide T_RM_ cells, while tumor-derived cells expressing these signatures comprise both T_RM_ and T_EX_ populations. Second, that tumor-derived cells expressing residency-associated signatures can be further segregated into T_EX_ and T_RM_ cells by the relative presence or absence of exhaustion-associated gene signatures. If bona fide T_RM_ cells exist in tumors, they would be expected to exhibit transcriptional similarity to T_RM_ cells in associated healthy tissue. To this end, we performed single-cell cellular indexing of transcriptomes and epitopes by sequencing (CITE-seq) with T cell receptor (TCR) profiling on CD3^+^ T cells from human BC tumors and normal breast tissue (Fig. 1i). CD8^+^ T cells from BC tumors and breast tissue were distributed over 15 clusters, classified into five main T cell subsets (T_EMRA_, T_EM_, mucosal-associated invariant T cells (MAIT), γδ T cells and CD103^+^ resident cells) based on protein and transcriptional profiles (Fig. 1j,k and Extended Data Fig. 1c). CD103^+^ ‘resident T cells’ shared expression of canonical residency genes including *ZNF683* (HOBIT) and *CXCR6*, and downregulation of *KLF2*, which controls principal tissue egress-promoting gene products^24^ (Fig. 1k). As above, we reasoned that tumor-derived CD103^+^ resident T cells would include both T_RM_ and T_EX_ populations whereas healthy tissue-derived cells would contain primarily T_RM_ cells. Accordingly, we defined two CD103^+^ clusters (c0, c5) as T_RM_ cells, based on their over-representation (>90%) within healthy tissue, whereas clusters found primarily in tumors and largely absent from healthy tissue (c7, c12, c15), were classified as T_EX_ cells (Fig. 1l,m). Pseudobulk principal component analysis (PCA) analysis confirmed the distinction between T_RM_ and T_EX_ clusters, while highlighting their similarity in the principal component (PC)1 axis, driven predominantly by the downregulation of genes associated with T cell egress including *KLF2* (Fig. 1n and Extended Data Fig. 1d).

Supporting our annotations, published T_RM_ gene signatures^2,21,28^ were enriched within both T_RM_ and T_EX_ populations whereas T_EX_ gene signatures^28^ were enriched selectively within T_EX_ cells (Fig. 1o). Segregation of T_EX_ and T_RM_ cell populations revealed that, although they share expression of CD103, CD69 and *ZNF683* (HOBIT), and the downregulation of *KLF2*, T_EX_ cells expressed higher levels of CD38, CD39, PD-1, CTLA4, *TIGIT* and *HAVCR2* (TIM3) (Extended Data Fig. 1e–i). These data show that T_EX_ cells within human tumors co-opt a residency program, and that commonly used T_RM_ cell-associated proteins or gene signatures cannot distinguish between T_RM_ and T_EX_ populations.

### T_RM_ and T_EX_ cells exhibit disparate functional capacities

**Fig. 2:**
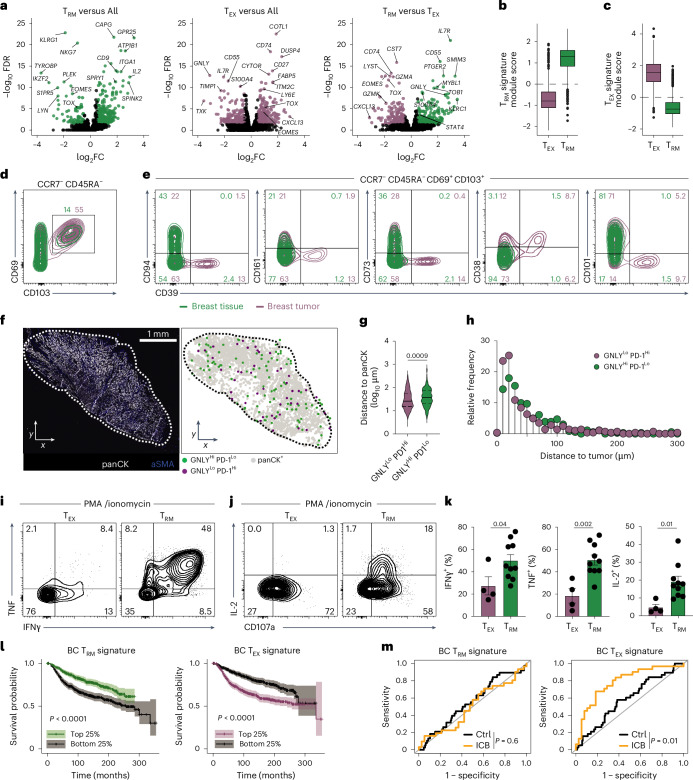
Spatial and functional characterization of CD103^+^ T_RM_ and T_EX_ cells in tumors. **a**, Volcano plots showing DE between T_RM_, T_EX_ and all other subsets in BC dataset. **b**,**c**, Enrichment of T_RM_ (**b**) and T_EX_ (**c**) gene signatures on labeled subsets. Box and whisker plots indicate minimum, maximum, median, and first and third quartiles. **d**,**e**, Flow cytometry, concatenated from *N* = 13 donors (*N* = 11 with tumor sample and *N* = 10 with healthy tissue). **d**, Expression of CD69 and CD103 on CCR7^−^CD45RA^−^ CD8^+^ T cells. **e**, Expression of T_RM_-defining and T_EX_-defining proteins on CD69^+^CD103^+^ T cells. **f**–**h**, CycIF imaging of BC tumors. **f**, Representative image of tumor section showing panCK (tumor) and aSMA (stroma) expression, and relative location of respective annotated cell types. Distance to nearest panCK^+^ cell (**g**), and frequency in bins (10 μm) segregated by distance of respective cell types to panCK^+^ cells (**h**), pooled from *N* = 7 donors (data in **g** analyzed by two-sided *t*-test). Expression of TNF, IFNγ (**i**), IL-2 and CD107a (**j**) on T_EX_ and T_RM_ cells from PMA-ionomycin stimulated CD8^+^ T cells from BC tumors or tissue as per clusters in Extended Data Fig. 3b. **k**, Summary of **i**–**j**. Donors contributing a minimum of ten cells within a cluster (T_RM_ or T_EX_ isolated from either tumor or healthy tissue) were enumerated and plotted (*N* = 4 T_EX_, *N* = 10 T_RM_), analyzed by two-sided *t*-test. Error bars, mean ± s.e.m. **l**, Survival of BC patients from the METABRIC dataset^58^ with the highest (top 25%) T_RM_ or T_EX_ gene signature score enrichments compared to patients with lowest (bottom 25%) gene enrichment scores, plotted on Kaplan–Meier curves with log-rank test. **m**, ROC curves from BC patients, either control (Ctrl, *N* = 210) or treated with ICB (pembrolizumab/anti-PD-1, *N* = 69) from the iSPY trial^31^. Clinical response to ICB associated with T_RM_ or T_EX_ gene signatures, respectively. Source data

**Extended Data Fig. 2:**
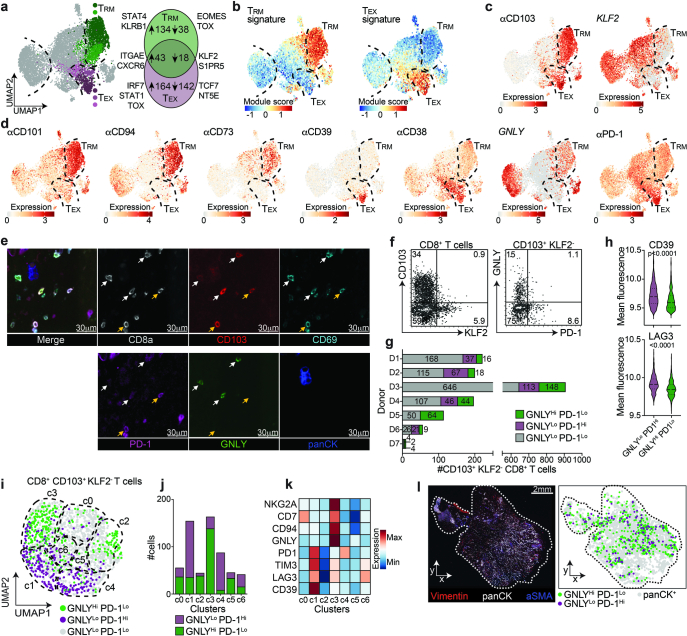
Lineage-defining genes and proteins enable in situ localization of T_RM_ and T_EX_*.* **a**, T_RM_ and T_EX_ signature genes. Shared genes indicate genes are up (or down)-regulated in the T_RM_ vs all *and* T_EX_ vs. all comparisons (Fig. 2a). T_RM_ (**b**) and T_EX_ (**c**) gene signature module scores displayed on BC UMAPs for summary module score data (Fig. 2b, c). **c-d**, Expression of respective genes and cell surface proteins displayed on BC UMAPs. **e-l**, CycIF imaging of BC tumours related to Fig. 2f–h. **e**, representative image of BC tumour using CycIF approach showing respective stains. Representative of 7 independent donors. **f**, Histocytometric gating strategy showing CD8^+^ T cells (CD3^+^ CD8^+^ CD4^−^) concatenated from CycIF images from 7 donors showing CD103, KLF2, GNLY, and PD-1 expression on respective populations. **g**, number of CD8^+^ CD103^+^ KLF2^−^ T cells identified in each slide from respective donors, broken down by GNLY and PD-1 expression based on gates in (f). **h**, Expression of CD39 and LAG3 proteins on respective populations, pooled from 7 donors. Two-sided Mann-Whitney test. **i**, UMAP projection and clustering of CD8^+^ CD103^+^ KLF2^−^ T cells, coloured by gates defined in (f). **j**, number of cells from respective gates identified within each cluster. **k**, relative expression of respective proteins in each cluster. **l**, representative image of tumour section showing panCK and aSMA expression, and relative location of respective annotated cell types.

To disentangle tumor-associated T_RM_ and T_EX_ cells, we developed gene signatures from our BC dataset that could distinguish these populations accurately. Genes were included in the T_RM_ gene signature based on differential expression (DE) in T_RM_ cells compared to other T cell subsets, followed by successive DE analysis between T_RM_ and T_EX_ metaclusters. An analogous approach was used to derive the T_EX_ gene signature (Fig. 2a–c and Extended Data Fig. 2a,b). Whereas both T_RM_ and T_EX_ cells were defined by CD103 expression and *KLF2* downregulation (Fig. 2d and Extended Data Fig. 2c), they were distinguished further by expression of markers including CD94, CD161, CD73, CD38, CD101, CD39, *GNLY* and PD-1 (Fig. 2e and Extended Data Fig. 2d). This enabled reliable discrimination of the two populations via cyclic immunofluorescence microscopy (CycIF^29,30^) and flow cytometry, allowing examination of their intratumoral location and functional properties.

Using a 47-marker CycIF panel, we identified CD103^+^ KLF2^−^ CD8^+^ T cells across seven BC patients (Extended Data Fig. 2e,f). These cells were stratified based on the relative expression of GNLY and PD-1 into GNLY^Hi^ PD-1^Lo^ and GNLY^Lo^ PD-1^Hi^ populations, approximating T_RM_ and T_EX_ cells, respectively (Extended Data Fig. 2e–g). GNLY^Lo^ PD-1^Hi^ cells expressed higher levels of CD39 and LAG3, consistent with an exhausted phenotype (Extended Data Fig. 2h). Unbiased clustering of CD103^+^ KLF2^−^ CD8^+^ T cells reinforced this distinction: GNLY^Hi^ PD-1^Lo^ cells were enriched in cluster C3, which expressed T_RM_-associated markers including CD94, CD7 and NKG2A, whereas GNLY^Lo^ PD-1^Hi^ cells dominated cluster C1 with increased TIM3, LAG3 and CD39 expression (Extended Data Fig. 2i–k), supporting our in situ gating strategy. Spatial analysis revealed that both populations localized near panCK^+^ tumor regions (Fig. 2f and Extended Data Fig. 2l). However, GNLY^Lo^ PD-1^Hi^ (approximating T_EX_) cells were, on average, significantly closer to panCK^+^ tumor cells, suggesting an increased potential for direct tumor interaction (Fig. 2g,h).

**Extended Data Fig. 3:**
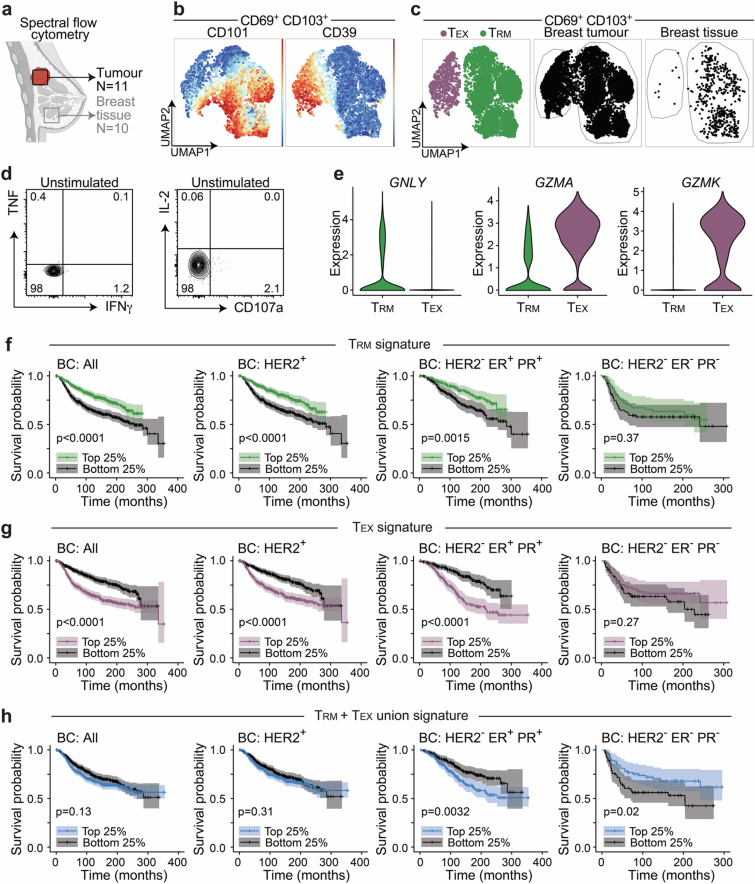
Functional assessment and survival associations of BC T_RM_ and T_EX_. **a-e**, Flow cytometry of PMA-ionomycin restimulated BC and breast tissue-derived CD103^+^ resident-phenotype T cells related to Fig. 2d–k. **a**, Schematic. **b-c**, CD69^+^CD103^+^CD45RA^−^CCR7^−^ CD8^+^ T cells represented in UMAP space based on the expression of CD69, CD101, CD39, CD94, CD73, CD103, CD45RA, CCR7, CD38 showing CD101 and CD39 expression (**b**) and annotated T_RM_ and T_EX_ populations and relative contribution by cells derived from BC tumours or tissue (**c**). **d**, Gating of TNF, IFNγ, IL-2, and CD107a for Fig. 2i–k based on unstimulated control. **e**, Expression of granulysin (*GNLY*), granzyme A (*GZMA*) and granzyme K (*GZMK*) on T_RM_ and T_EX_ populations from BC CITEseq dataset. **f-h**, Survival of BC patients from the dataset with the highest (top 25%) T_RM_ (**f**), T_EX_ (**g**), or combined T_RM_ + T_EX_ union (**h**) gene signature score enrichments compared to patients with lowest (bottom 25%) gene enrichment scores and plotted on Kaplan-Meier curves with log-rank test, as in (Fig. 2l), segregated by BC subtypes (as annotated).

Beyond phenotypic and spatial differences, we also observed distinct functional capacities between T_RM_ and T_EX_ populations. Upon ex vivo restimulation, T_RM_ cells exhibited higher production of interferon gamma (IFNγ), tumor necrosis factor (TNF) and interleukin (IL)-2 and showed elevated expression of granulysin (*GNLY)*, while T_EX_ cells expressed more granzyme A (*GZMA*) and granzyme K (*GZMK*) (Fig. 2i–k and Extended Data Fig. 3a–e). Moreover, deconvolution of these populations in transcriptomic datasets revealed that enrichment for the T_RM_ gene signature was associated with improved overall survival in BC patients, whereas T_EX_ gene signature enrichment correlated with poorer outcomes (Fig. 2l). This association appeared BC-subtype specific, given that triple-negative BC (TNBC) survival was best predicted by total CD103^+^ (both T_RM_ and T_EX_) cells, consistent with our previous work (Extended Data Fig. 3f–h)^11^. Further, we tested the association of the T_RM_ and T_EX_ signatures with responses to ICB (pembrolizumab/αPD-1) in the I-SPY2 trial^31^, revealing that patients with higher T_EX_ gene signature expression were more likely to achieve a pathologic complete response (pCR) following ICB, whereas elevated T_RM_ signature expression was inconsequential to ICB responsiveness (Fig. 2m). These data indicate that, although T_RM_ cells are associated with positive prognoses in BC, current ICB therapies exclusively target and enhance T_EX_ cell-mediated anti-tumor responses.

### T_RM_ and T_EX_ gene signatures delineate T_RM_ cells across tumors

**Fig. 3:**
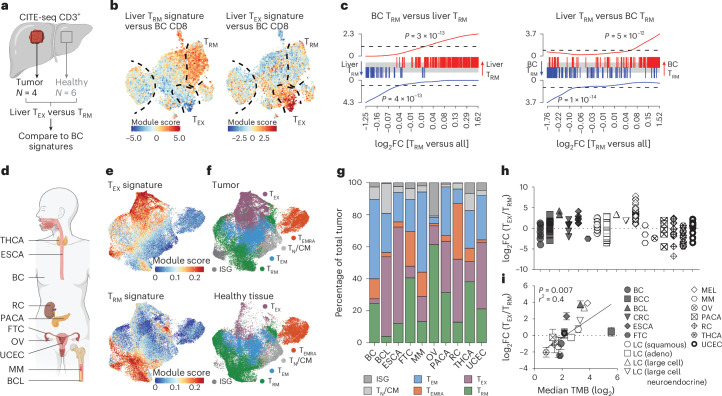
Specific T_RM_ and T_EX_ gene signatures enable T_RM_ and T_EX_ cell identification across tumors. **a**, CITE-seq of CD3^+^CD8^+^ T cells isolated from secondary liver tumors (CRC patients, *N* = 4) and noncancerous liver tissue (*N* = 6). Liver T_RM_ versus T_EX_ gene signatures were acquired as described for BC signatures. **b**, Overlay of liver T_RM_ and T_EX_ signature module scores on BC dataset. **c**, Gene set enrichment analysis (GSEA) of BC T_RM_ versus liver T_RM_ signatures and vice versa. Barcode plots show GSEA using a running-sum statistic; *P* value determined by permutation testing. **d**–**g**, scRNA-seq of tumor-associated CD8^+^ T cells from various cancers. **d**, Cancers from pancancer atlas^28^. BCC, basal cell carcinoma; FTC, fallopian tube cancer; LC, lung cancer; MM, multiple myeloma; OV, ovarian cancer; PACA, pancreatic cancer; RC, renal cancer; THCA, thyroid cancer; UCEC, uterine corpus endometrial cancer. **e**, Overlay of refined T_EX_ (top) and T_RM_ (bottom) gene signatures on CD8^+^ T cells on UMAP of pancancer scRNA-seq dataset^28^. **f**, Tumor-derived (top) and healthy tissue-derived (bottom) CD8^+^ T cells and annotated clusters. **g**, Relative frequencies of different CD8^+^ T cell subsets by cancer^28^. **h**, The log_2_ ratio of T_EX_ to T_RM_ across different cancers^28,32–37^. **i**, T_EX_/T_RM_ ratio association with median TMB^38^. Error bars, mean ± s.e.m. *P* value indicates that the slope of the regression line departs from zero. Illustrations in **a** and **d** created using BioRender.com. Source data

**Extended Data Fig. 4:**
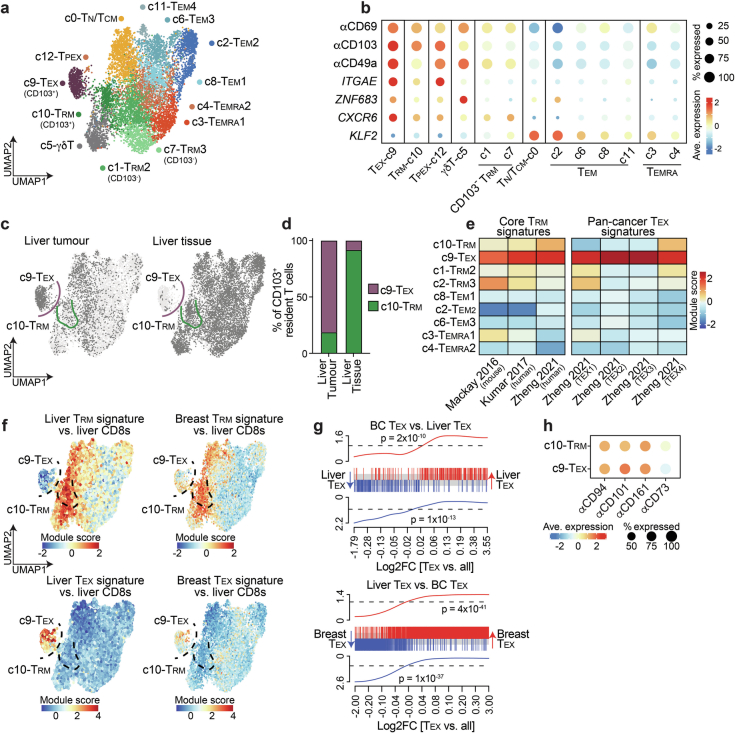
Deconvolution of CD103+ T_RM_ and T_EX_ cells in liver metastases. **a-d**, CITEseq of CD3^+^CD8^+^CD4^−^ non-MAIT cells isolated from secondary liver tumours (colorectal cancer patients, N=4) and non-cancerous liver tissue (N=6) as depicted in Fig. 3a. **a**, Data was Harmony integrated, and unified protein and RNA-seq data represented on weighted nearest neighbours UMAP, coloured by clusters that were annotated based on expression of lineage-defining cell surface proteins and genes. **b**, Expression of selected cell surface proteins (αCD69, αCD103,) and genes (*ITGAE, ZNF683, CXCR6, KLF2*) on respective clusters. **c-d**, CD8^+^ T cells segregated by tissue of origin (**c**), and relative cluster composition of CD103^+^ resident T cells (c9+c10) isolated from BC tumours or tissue (**d**). **e**, Average module scores of published T_RM_^2,21,28^ and T_EX_^28^ cell gene signatures by annotated clusters. **f**, Module score overlays of relevant signatures on liver CITEseq dataset. **g**, gene-set enrichment analysis of BC T_EX_ vs liver T_EX_ signatures and vice versa. Barcode plots show GSEA enrichment using a running-sum statistic, p-val determined by permutation testing. **h**, Expression of respective cell surface proteins across annotated clusters.

To determine the applicability of these BC T_RM_ and T_EX_ gene signatures across tumor types, we next developed signatures from CD8^+^ T cells isolated from liver tumors for comparison. To this end we performed CITE-seq on CD8^+^ T cells isolated from liver metastases from colorectal cancer (CRC) patients, paired noncancerous liver tissue, and liver tissue from cancer-free donors (Fig. 3a). Two CD103^+^ resident (CD69^+^, CD103^+^, *KLF2*^−^) T cell clusters were identified and denoted as T_EX_ and T_RM_ populations as described above, with T_EX_ cells mostly present in tumor-derived tissue and T_RM_ clusters present in both tumor and noncancerous tissue (Extended Data Fig. 4a–d). In line with our findings in BC, we found that both T_RM_ and T_EX_ populations were enriched for published T_RM_ but not T_EX_ gene signatures (Extended Data Fig. 4e). Liver T_RM_ and T_EX_ signatures could accurately identify BC T_RM_ and T_EX_ cells (Fig. 3b) and, similarly, BC T_RM_ and T_EX_ signatures identified liver T_RM_ and T_EX_ cells, respectively (Extended Data Fig. 4f). Overall, BC T_RM_ signature genes were highly enriched in liver T_RM_ cells (and vice versa), which was also true for the respective T_EX_ gene signatures highlighting shared gene expression across tumor-associated T_RM_ and T_EX_ cells from disparate tumor types (Fig. 3c and Extended Data Fig. 4g).

**Extended Data Fig. 5:**
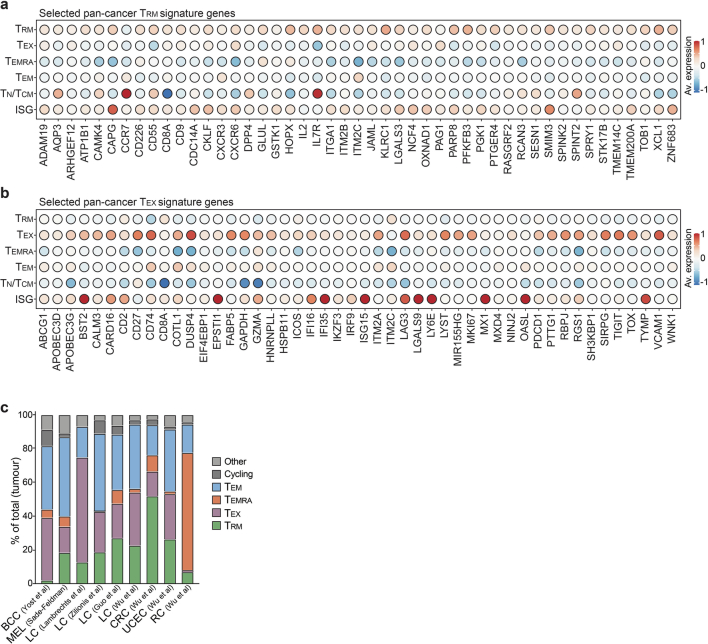
Pan-cancer T_RM_ and T_EX_ signature genes. **a-b**, Relative expression of the top genes from pan-cancer T_RM_ (**a**) and T_EX_ (**b**) gene signatures across respective CD8^+^ T cell populations in the pan-cancer atlas^28^. **c**, Relative frequencies of different CD8^+^ T cell subsets by cancer from respective datasets^32–37^.

Whereas the collection of genes within T_RM_ and T_EX_ signatures correlated strongly across BC and liver tumors, the expression of individual genes and surface proteins was not universally consistent. For example, CD94, CD101, CD161 and CD73 were enriched specifically in T_RM_ cells in BC (Fig. 2e) but not in liver-derived tumors (Extended Data Fig. 4h). Therefore, caution must be applied when extrapolating individual genes and proteins across T cell populations in distinct settings. Nonetheless, by focusing on the leading-edge genes (Fig. 3c and Extended Data Fig. 4g), we defined broadly applicable pancancer T_RM_ and T_EX_ signatures (Extended Data Fig. 5a,b and Supplementary Table 2). Using these gene signatures, we could distinguish T_RM_ and T_EX_ cells across a range of tumors from several datasets, including a pancancer atlas^28^ and additional studies^32–37^ (Fig. 3d–g and Extended Data Fig. 5c). A composite of the samples from the pancancer atlas showed that T_EX_ cells were detected predominantly in tumor-derived tissue and largely absent from noncancerous, healthy tissue (Fig. 3e,f). When analyzed by tumor type, the relative abundance of T_EX_ and T_RM_ cells varied, with melanoma (MEL), esophageal cancer (ESCA) and B cell lymphoma (BCL) displaying the highest T_EX_ to T_RM_ ratio among the cancers examined (Fig. 3g,h). This ratio correlated positively with tumor mutational burden (TMB)^38^, indicating that neoantigen load may preferentially promote T_EX_ cell formation (Fig. 3i). Overall, these data demonstrate our ability to deconvolute T_RM_ and T_EX_ cells across various tumor settings, facilitating a detailed investigation of their functional and developmental differences.

### Tumor-associated T_RM_ are clonally distinct and enriched for virus-specific cells

**Fig. 4:**
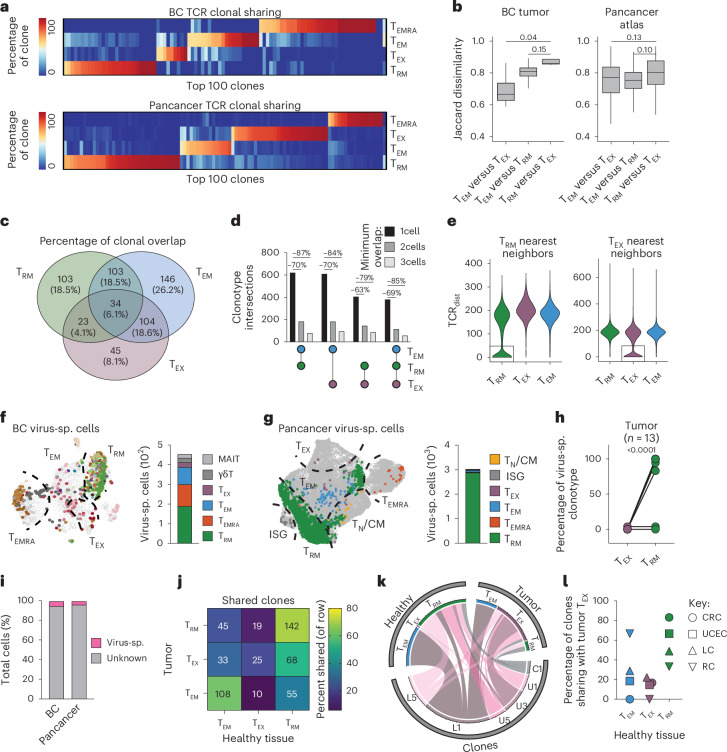
Tumor-associated T_RM_ and T_EX_ cells are clonally distinct with discrete specificities. **a**, Relative frequency of the top 100 expanded TCR clones within each metacluster from the BC dataset (top) or pancancer atlas (bottom)^28^. **b**, Jaccard dissimilarity index scores (1 − Jaccard index) for expanded (minimum two cells) tumor-derived T cell clones showing dissimilarity between respective populations from BC dataset or pancancer atlas, analyzed by two-sided *t*-test. Box and whisker plots indicate minimum, maximum, median, and first and third quartiles. **c**, Venn diagram indicating clonal overlap between expanded T_RM_, T_EM_ or T_EX_ clonotypes from BC dataset. **d**, UpSet plot showing the overlap among T_EM_, T_RM_ and T_EX_ cell types from pooled BC, liver and pancancer datasets, with minimum overlap cutoffs of one, two or three cells. **e**, Violin plots representing TCR_dist_ analysis of pooled BC, liver and pancancer datasets, showing the distance between T_RM_ or T_EX_ with respective subsets, where lower values indicate greater similarity. **f**,**g**, Distribution and enumeration of cells expressing virus-specific TCRs as determined by VDJdb^41–43^ in the BC (**f**) and pancancer (**g**) datasets. Individual dots indicate virus-specific cells, uniquely colored by clone (**f**) or phenotype (**g**). **h**, Frequency of virus-specific cells within each clonotype that adopt T_RM_ or T_EX_ cell phenotypes, analyzed using a two-sided paired *t*-test. **i**, Frequency of virus-specific T cells of total CD8^+^ T cells in respective datasets. **j**–**l**, Clonal sharing between tumor and healthy tissue-derived CD8^+^ T cells^36^. **j**, Clonal sharing between subsets detected across tumor versus healthy tissue-derived CD8^+^ T cells, filtered on clones identified in both tissues. Heatmap scaled by percentage sharing in each row, numbers indicate shared clones pooled from CRC, UCEC, LC and RC^36^. **k**, Circos plot indicating selected clones from distinct cancers, and number of cells from each clonotype occupying each tissue and phenotype. **l**, Summary of clonal sharing between tumor T_EX_ and respective phenotype in healthy tissue, split by cancer. Source data

**Extended Data Fig. 6:**
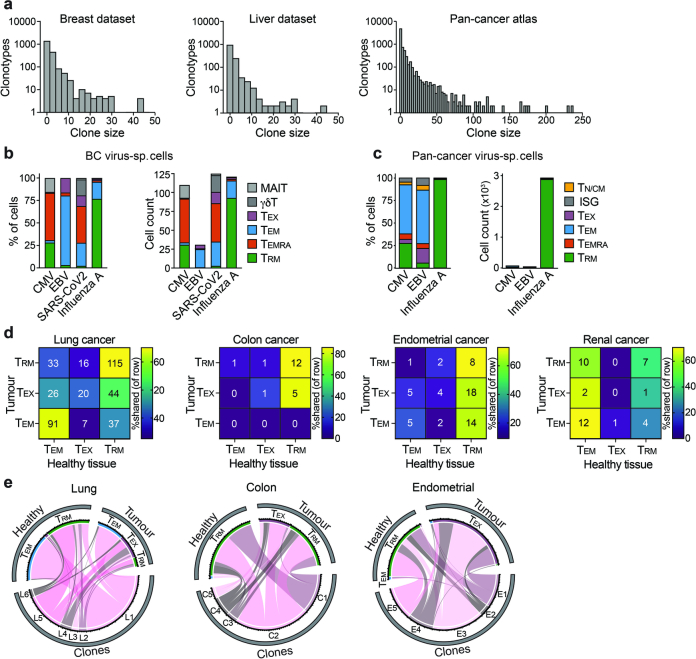
Assessment of virus-specific clones and TCR sharing across tissues. **a**, Clone size distribution across respective datasets. **b-c**, Frequency and count of cells from BC dataset (b) and pan-cancer atlas (c) split by respective virus-specificity. **d-e**, Clonal sharing between tumour and healthy tissue-derived CD8^+^ T cells^36^ related to Fig. 4j–l. Clonal sharing between subsets detected across tumour vs healthy tissue-derived CD8^+^ T cells, filtered on clones identified in both tissues. **d**, Heatmap scaled by % sharing in each row, split by respective cancers^36^. **e**, Circos plots indicating selected clones from respective cancers, and number of cells from each clonotype occupying each tissue and phenotype.

Given the correlation between T_EX_ cell abundance and TMB, we hypothesized that T_EX_ and T_RM_ populations may be clonally distinct, reflecting differences in antigen specificity. We therefore examined clonal overlap among the top 100 expanded TCR clones across T cell subsets in both our BC dataset and the pancancer atlas. In both datasets, T_RM_ and T_EX_ cells displayed limited clonal overlap with each other and instead showed greater clonal overlap with T_EM_ cells (Fig. 4a). High Jaccard dissimilarity scores supported this, indicating that TCR repertoires of tumor-derived T_RM_, T_EX_ and T_EM_ cells were largely distinct (Fig. 4b). Within our BC dataset, expanded clones were shared occasionally across subsets, with most sharing occurring between T_RM_ and T_EM_ or T_EX_ and T_EM_, rather than directly between T_RM_ and T_EX_ (Fig. 4c). Since no public TCRs were present across donors in our BC dataset, liver dataset or the pancancer atlas, we pooled the TCR data for further examination. We assessed clonotype sharing among T_RM_, T_EX_ and T_EM_ cells at various thresholds based on overlapping cell numbers for each clonotype, and found that most clonal overlap among T_RM_, T_EX_ and T_EM_ cells was due to a single shared cell despite substantial numbers of expanded clones (Fig. 4d and Extended Data Fig. 6a). These data indicate that, while a single clone can adopt T_RM_, T_EX_ or T_EM_ phenotypes, there is preferential development of one subset for a given TCR.

Structurally similar TCRs are predicted to recognize similar epitopes. Using TCRdist^39,40^, we identified clear structural segregation between T_RM_ and T_EX_ cell TCRs, with substantial structural similarity predicted only among cells of the same phenotype. This suggests that T_RM_ and T_EX_ populations possess distinct epitope specificities (Fig. 4e). To investigate further, we integrated TCR sequences and human leukocyte antigen (HLA) allele expression with VDJdb^41–43^ to predict viral reactivity of CD8^+^ T cells in both our BC dataset and the pancancer atlas. Predicted virus-specific clonotypes were associated predominantly with a T_RM_ phenotype, whereas virus-specific T_EX_ cells were exceedingly rare (Fig. 4f–h). The propensity to adopt a T_RM_ cell phenotype varied depending on viral specificity. Predicted influenza A-specific cells most frequently exhibited a T_RM_ phenotype, whereas EBV-specific T_RM_-like cells were rarely identified (Extended Data Fig. 6b,c).

Critically, only a small fraction of total CD8^+^ T cells in both datasets was predicted to be virus-specific (Fig. 4i), yet these tumor-antigen-independent cells were enriched for the T_RM_ gene signature. Previous studies have shown that tumor-antigen-specific cells express markers such as CD39 (refs. ^44,45^), which are characteristic of T_EX_ cells in our classification. We therefore hypothesized that tumor-derived clones with a T_EX_ gene signature, when detected in healthy tissue where tumor antigen is presumed absent, would instead be enriched for the T_RM_ gene signature.

Given clonal sharing between tumor and healthy tissue-derived cells in our BC and pancancer datasets was limited, probably due to inadequate sampling, we leveraged a dataset in which T cell clones were identified in both tumor and adjacent healthy tissue^36^. We extracted CD8^+^ T cell clones shared across sites and annotated them based on our T_RM_ and T_EX_ gene signatures. Consistent with our hypothesis, there was significant clonal sharing between tumor-derived T_EX_ and healthy tissue-derived T_RM_ cells (Fig. 4j,k and Extended Data Fig. 6d,e). Indeed, when clonal overlap was observed, tumor-derived T_EX_ clonotypes were shared more frequently with healthy T_RM_ than with other T cell types (Fig. 4l). Together, these data indicate that the T_RM_ gene signature is enriched in predicted tumor-antigen-independent memory T cells, and tumor-specific cells in healthy noncancerous tissues.

### Antigen drives the distinction between T_RM_ and T_EX_ cells in tumors

**Fig. 5:**
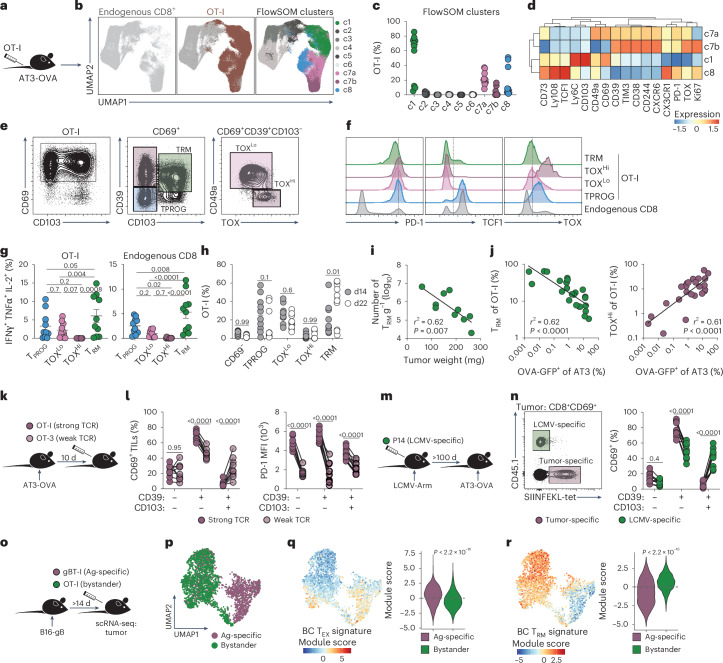
Low-avidity and bystander CD8^+^ T cells preferentially adopt a T_RM_ phenotype in tumors. **a**–**f**, Mice received 1 × 10^4^ naive OT-I T cells and were challenged with AT3-OVA; TILs were analyzed on day 22. **a**, Experimental schematic. **b**, UMAP of CD8⁺ TILs (defined by markers in Extended Data Fig. 7a) showing OT-I T cells and FlowSOM clusters; Cluster 7 was stratified by TOX expression (into c7a (TOX^Lo^) and c7b (TOX^Hi^)). **c**, Distribution of OT-I T cells across FlowSOM clusters. **d**, Relative marker expression by cluster. Representative gating (**e**) and marker expression (**f**) from tumor-derived OT-I T cells. **g**, Cytokine expression post-PMA/ionomycin restimulation. Error bars, mean ± s.e.m. **h**, OT-I frequencies at days 14 and 22. **i**, T_RM_ cell number per gram tumor versus tumor mass at day 14. **j**, Proportion of T_RM_ and TOX^Hi^ OT-I versus %GFP-OVA^+^ AT3 at day 22. **k**,**l**, Effector OT-I (strong TCR signaling) and OT-3 (weak TCR signaling) T cells were cotransferred into tumor-bearing mice (day 10); intratumoral T cells were analyzed on day 28. **k**, Schematic. **l**, Frequency of CD69⁺ cells and PD-1 expression. **m**,**n**, LCMV-immune mice (receiving 5 × 10⁴ P14 before infection) were challenged with AT3-OVA >100 days post-LCMV infection; intratumoral T cells analyzed on day 23 post-tumor inoculation. **m**, Schematic. **n**, Frequencies of CD45.1⁺ LCMV-specific (P14) versus SIINFEKL-tetramer⁺ tumor-specific cells among CD8⁺CD69⁺ TILs. **o**–**r**, scRNA-seq of bystander (OT-I) and Ag-specific (gBT-I) CD8⁺ T cells cotransferred into mice with B16-gB melanoma tumors. **o**, Schematic. **p**, UMAP of sorted cells colored by transgenic T cell. **q**,**r**, Overlay of BC T_EX_ (**q**) and T_RM_ (**r**) gene signatures. *P* values from two-sided Wilcoxon signed-rank test. Statistics: **b**–**j**,**l**, pooled from two independent experiments; **n**, representative of two experiments, *N* = 10 mice; **b**–**f**, representative of more than eight total experiments, minimum *N* = 5 mice per group per experiment; **g**, one-way repeated-measures ANOVA with Tukey post-test; **h**, two-way ANOVA with Sidak test; **i**,**j**, *r*^2^ indicates fit of linear regression line and *P* value indicates slope departs from zero. **l**,**n**, Two-way repeated-measures ANOVA with Sidak test. **l**,**n**, Connected points from individual mice. Panels **a**, **k**, **m** and **o** adapted from ref. ^47^, Springer Nature America. Source data

**Extended Data Fig. 7:**
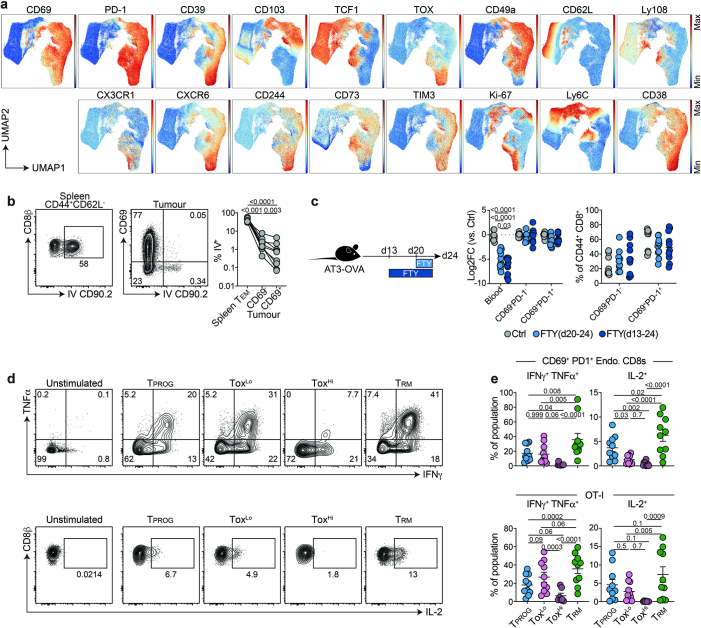
Phenotypic and functional assessment of AT3-OVA TIL populations. **a**, Expression of respective proteins on CD8^+^ T cells isolated from AT3-OVA tumours projected in UMAP space, related to Fig. 5a–d. **b**, Gating and intravenous (IV) labelling of respective CD8^+^ T cell populations from spleen and tumour at d24 post-inoculation. **c**, FTY720 treatment of AT3-OVA bearing mice showing relative cell number (normalised to median of control group in each experiment) and frequency of CD69^+^ PD-1^+^ cells within CD44^+^ CD8^+^ T cells from tumours, and total CD8^+^ T cells from the blood. **d**, representative gating of IFNγ, TNFα, and IL-2 on respective populations (as in Fig. 5e). Gates based on unstimulated control. **e**, % expression of IFNγ, TNFα, and IL-2 on respective endogenous CD8^+^ (top) and OT-I (bottom) T cell populations. Statistics: **a-e**, pooled from 2 independent experiments, minimum N=5 mice/group/experiment. **b**, one-way repeated-measures ANOVA with Holm-Sidak test, connected points from individual mice. **c**, Two-way ANOVA with Tukey’s test. **e**, one-way repeated-measures ANOVA with Tukey post-test. Panel **c** adapted from ref. ^47^, Springer Nature America.

Given that putative tumor-antigen-specific T cells preferentially adopt a T_RM_ phenotype in surrounding tissues where tumor antigen may be absent, we hypothesized that tumor-antigen reactivity drives the divergence between tumor-associated T_RM_ and T_EX_ cells. To test whether tumor-specific T_RM_ can form in tumors, we used an orthotopic murine BC (AT3) line expressing the model antigen ovalbumin (OVA). High-dimensional flow cytometry of tumor-infiltrating T cells (TILs) revealed four clusters enriched for tumor-specific OT-I T cells expressing CD69 and PD-1, and differing in markers such as CD39, CD103, TCF1, TOX and TIM3 (Fig. 5a–f and Extended Data Fig. 7a). We annotated these subsets based on phenotypic and functional characteristics. First, we defined resident cells as nonmigratory, using indirect indicators of tissue residency, including intravenous (IV) labeling and FTY720 treatment. Over 90% of TILs were IV-negative, and CD69^+^ TILs showed reduced IV-staining relative to CD69^−^ TILs (Extended Data Fig. 7b). TIL frequencies were also unaffected by FTY720 treatment (Extended Data Fig. 7c), supporting that most TIL are resident.

**Extended Data Fig. 8:**
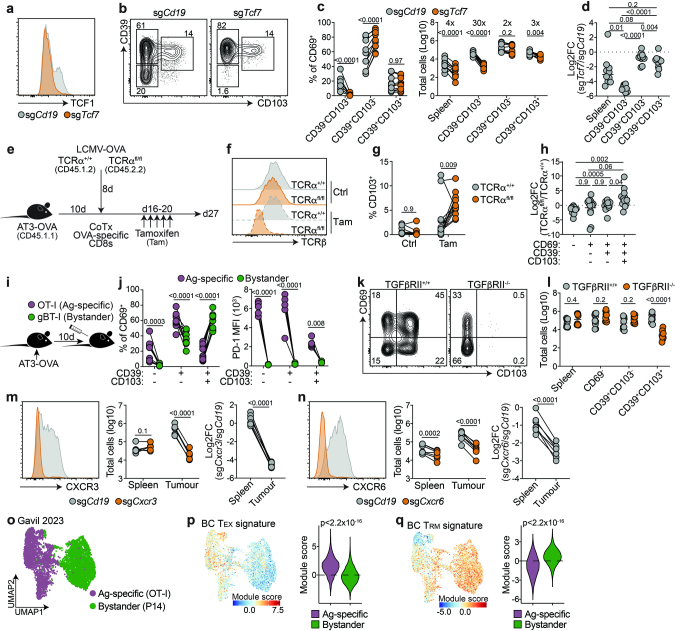
Mechanistic assessment of factors controlling formation of TIL populations. **a-d**, CRISPR/Cas9 depletion of *Tcf7* from naïve OT-I T cells in AT3-OVA tumours harvested d24 post-inoculation. **a**, TCF1 expression on co-transferred sg*CD19* (control) and sg*Tcf7* treated OT-I T cells. **b**, Representative gating of CD69^+^ OT-I T cells. **c**, frequency and number of CD69^+^ OT-I T cells. **d**, Log2 fold-change of cell numbers. **e-f**, Impact of TCRα depletion on TIL populations. Congenically distinct TCRα^+/+^ Cre^ERT2^ and TCRα^fl/fl^ Cre^ERT2^ mice were infected with LCMV-OVA; SIINFEKL-tetramer^+^ CD8^+^ T cells were sorted from spleens at d8 post-infection and co-transferred into AT3-OVA bearing mice 10 d post-tumour. Tamoxifen (Tam) or vehicle control was given on d16-20 post-tumour, and harvested on d27. **e**, schematic. **f**, TCRβ staining. **g**, % CD103 expression. **h**, ratio of cell counts. **i-j**, Effector OT-I (Ag-specific) and gBT-I (bystander) CD8^+^ T cells were co-transferred into mice 10 d post-AT3-OVA inoculation, analysed 7 d later. **i**, Schematic. **j**, Frequency and PD-1 expression of CD69^+^ populations. **k-l**, Effector control (TGFβRII^+/+^) and TGFβRII^−/−^ bystander OT-I T cells transferred into AT3 tumour-bearing mice 10 d post inoculation. **k**, gating, **l**, cell counts 6 d following transfer. **m-n**, Effector control (sg*CD19*) and CXCR3 (m, sg*CXCR3*) or CXCR6 (n, *sgCXCR6*) deficient gBT-I T cells co-transferred into AT3-OVA tumour bearing mice 10 d post-inoculation; spleen and tumours analysed 7 d later. Expression of CXCR3 or CXCR6, cell counts, and ratios of cells. **o-q**, Analysis of CITEseq of LCMV-specific P14 or tumour-specific OT-I T cells isolated from EO771-OVA BC tumours^10^. **o**, Unified cell-surface protein and RNA-seq expression data represented on weighted nearest neighbours UMAP coloured by T cell transgenic. **p-q**, quantification of BC T_EX_ (**p**) and T_RM_ (**q**) signature module scores, p-value calculated by two-sided Wilcoxon signed-rank test. Statistics: **a-d**, i-l, pooled from 2 independent experiments, N=5 mice per experiment; m-n, representative of 2 independent experiments with a minimum of 5 mice per group. **c,g,j,m,n**, linked symbols indicate cells from the same mouse, 2-way repeated-measures ANOVA with Sidak post-test. **d,h**, 1-way repeated-measures ANOVA with Tukey post-test. **l**, 2-way ANOVA with Sidak post-test. p-q, Wilcoxon signed-rank test. Panels **e** and **i** adapted from ref. ^47^, Springer Nature America.

Second, we considered cells exhausted if they exhibited functional impairment, and memory cells as those capable of persisting without ongoing antigen stimulation. Accordingly, CD39^+^ CD103^+^ cells were annotated as T_RM_ cells based on superior functionality, whereas CD39^+^ CD103^−^ TOX^Hi^ cells represented the most dysfunctional, exhausted population (Fig. 5g and Extended Data Fig. 7d,e). A separate CD69^+^ CD39^−^ CD103^−^ population expressed TCF1, PD-1 and TOX, consistent with progenitor exhausted T cells (T_PROG_), which were depleted selectively when *Tcf7* expression was ablated (Fig. 5e,f and Extended Data Fig. 8a–d). The number of T_RM_ phenotype cells correlated inversely with tumor size at early timepoints (d14) (Fig. 5i), and acquisition of the T_RM_ phenotype correlated with the loss of OVA–GFP expression from tumor cells over time (d23) (Fig. 5j). In contrast, TOX^Hi^ exhausted cells were enriched in tumors that maintained high tumor-antigen load, consistent with the role of antigen in driving T cell exhaustion (Fig. 5j). Inducible TCR depletion in tumor-specific T cells increased both the frequency and number of CD103^+^ T cells (Extended Data Fig. 8e–h), further supporting that CD103 expression marks memory T cells in this model, and that tumor-specific T_RM_ cells form or persist preferentially when cognate antigen is absent, consistent with classical T cell memory paradigms.

Given that intratumoral TCR signaling appeared to influence T_EX_ versus T_RM_ cell fate, we next assessed whether reducing TCR signal strength would also influence this distinction. Indeed, OT-3 T cells, which have reduced TCR signaling compared to OT-I T cells, displayed increased CD103 and decreased PD-1 expression (Fig. 5k,l), indicating that low-avidity TCR signaling promotes T_RM_ cell differentiation within tumors. Consistent with the T_RM_ phenotype of virus-specific T cells in human tumors, virus-specific memory T cells generated >100 days after LCMV infection infiltrated AT3-OVA tumors and adopted a T_RM_ phenotype (Fig. 5m,n). Transfer of tumor-specific (OT-I) and nonspecific bystander (gBT-I) effector T cells (T_EFF_) into tumor-bearing mice showed that bystander T cells acquired a T_RM_ phenotype more readily (Extended Data Fig. 8i,j). These bystander T cells expressed significantly lower levels of T_EX_-related proteins including PD-1 (Extended Data Fig. 8j). The development of intratumoral bystander T_RM_ cells required intrinsic TGFβ-signaling (Extended Data Fig. 8k,l), and their efficient tumor entry was dependent on CXCR3 and CXCR6 expression (Extended Data Fig. 8m,n).

We next speculated that the distinct transcriptional T_EX_ and T_RM_ cell signatures derived from patient samples above may correlate similarly with tumor-antigen reactivity. To assess this, we performed scRNA-seq on tumor-specific (gBT-I) and bystander (OT-I) T cells derived from B16-gB tumors and observed discrete clustering of these populations (Fig. 5o,p). Notably, tumor-specific T cells displayed enrichment of the human BC T_EX_ gene signature (Fig. 5q), whereas bystander T cells exhibited high expression of the BC T_RM_ gene signature (Fig. 5r). Consistently, bystander T cells from E0771 BC tumors^10^ expressed the T_RM_ gene signature and tumor-specific T cells expressed the T_EX_ gene signature (Extended Data Fig. 8o–q). Thus, the relative presence or absence of intratumoral TCR signaling drives the distinct transcriptional profiles of tumor-associated T_RM_ and T_EX_ cells. Altogether, these data indicate that strong and persistent TCR signaling is antithetical to T_RM_ cell development, suggesting that bona fide T_RM_ cells within human tumors are probably tumor-agnostic bystanders, low-affinity tumor-specific T cells or T cells not in contact with their cognate antigen.

### Tumor antigen drives T_RM_ cells towards a T_EX_ cell fate

**Fig. 6:**
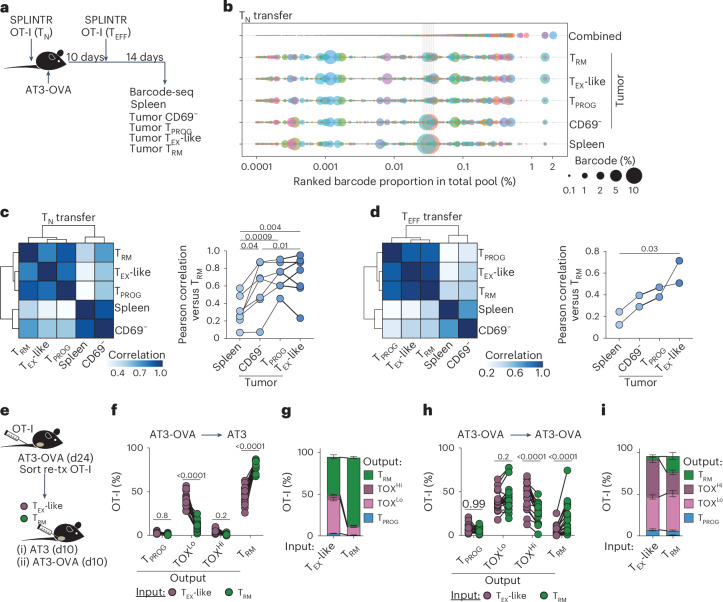
Tumor-associated T_RM_ cells can be driven towards exhaustion. **a**–**d**, SPLINTR barcode-seq of naive (T_N_) or T_EFF_ OT-I T cells transferred into mice AT3-OVA tumor-bearing mice. **a**, Schematic. TIL populations as defined in Fig. 5e. T_EX_-like population includes TOX^Lo^ and TOX^Hi^ populations. **b**, Barcode representation in a T_N_ transferred mouse sorted by total pooled barcode where bubble size reflects clone proportion in sample. **c**,**d**, Pearson correlation of barcodes identified in respective populations from representative mouse (heatmaps) and all repeats compared to T_RM_ population (line-plot) from T_N_ (**c**) or T_EFF_ (**d**) transfer. **e**–**i**, Sort-retransfer of respective OT-I populations from AT3-OVA mice. **e**, Schematic. Sorted cells were cotransferred intratumorally into recipient mice. **f**,**g**, Percentage of populations isolated from AT3-bearing recipient mice, split by input cell phenotype and output phenotype in recipient mouse, showing independent mice (**f**) and summary (**g**). **h**,**i**, Percentage of populations isolated from AT3-OVA bearing recipient mice, split by input cell phenotype, and output phenotype in recipient mouse, showing independent mice (**h**) and summary (**i**). Statistics: **b**,**c**, representative of eight independent biological replicates from three experiments; one-way repeated-measures ANOVA with Holm–Sidak post-test; **d**, representative of two independent biological repeats each with technical replicates; one-way repeated-measures ANOVA with test for linear trend. **g**,**i**, Error bars, mean ± s.e.m.; **f**–**i**, pooled from two independent experiments, *N* = 19 total mice; two-way repeated-measures ANOVA with Sidak post-test. Panels **a** and **e** adapted from ref. ^47^, Springer Nature America. Source data

The observation that T_RM_ and T_EX_ cells are clonally distinct in human tumors probably reflects intrinsic differences in antigen reactivity, rather than indicating that these populations derive from separate precursor cells. Notably, this clonal distinction does not preclude the presence of tumor-specific T_RM_ cells, nor the possibility of clonal overlap with T_EX_ cells. Our analyses suggested that such clonal overlap could occur when tumor-specific T_RM_ cells are present in tumors or in tissues where cognate antigen is presumably absent. These results did not resolve whether tumor-specific T_RM_ and T_EX_ cells arise from a shared progenitor or from distinct developmental lineages. To address this, we employed the SPLINTR barcoding system^46^ to introduce unique barcodes into mono-specific naive or T_EFF_ OT-I T cells before adoptive transfer into AT3-OVA tumor-bearing mice (Fig. 6a). Naive barcoded T cells were generated by transducing OT-I hematopoietic stem cells with SPLINTR lentivirus, followed by intrathymic injection into sublethally irradiated recipients. After 8 weeks, naive T cells pooled from 20 chimeric donors were transferred (2 × 10³ or 1 × 10⁴) into AT3-OVA-bearing mice. At 24 days postinoculation, SPLINTR-barcoded OT-I T cells were sorted from spleens and tumors (CD69^−^, T_PROG_, T_RM_ and CD39⁺CD103^−^ ‘T_EX_-like’ subsets) and barcode distribution assessed by DNA sequencing.

**Extended Data Fig. 9:**
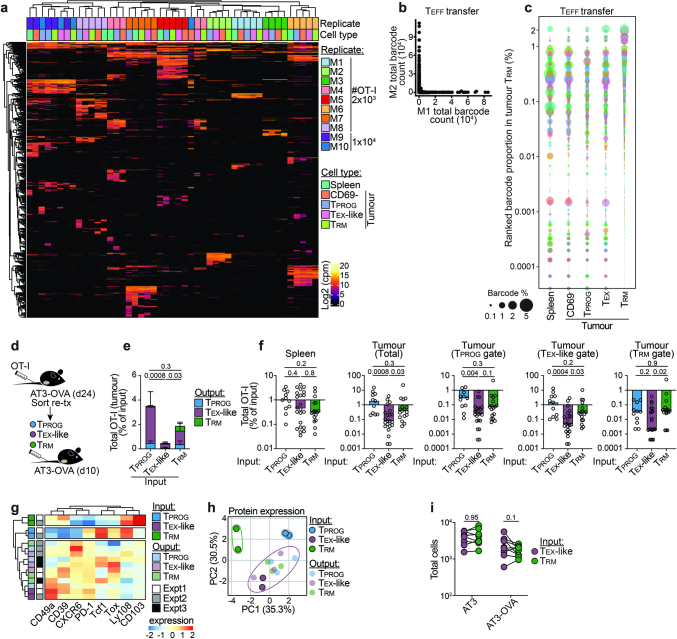
Developmental relationship between intratumoural T_RM_ and T_EX_. **a-c**, Related to Fig. 6a–d. **a**, heatmap showing counts of individual barcodes (y-axis) across different replicate mice and sorted populations from naïve OT-I SPLINTR experiments. **b**, Total barcode-seq counts of each identified barcode from two (M1 vs M2) effector OT-I SPLINTR experimental replicates. **c**, Barcode-seq data from representative effector OT-I SPLINTR experiment (M1) sorted by frequency in the tumour T_RM_ sample, where bubble size reflects clone proportion in sample. **d-h**, Sort and intravenous retransfer of AT3-OVA intratumoural populations. **d**, Schematic. **e**, Enumeration of transferred populations 28 d post-tumour inoculation as the frequency of input cell number. Error bars indicate mean +/- SEM. **f**, Cell numbers of respective populations isolated from spleen and tumours, normalised to input frequency, isolated 28 d post-tumour inoculation. Bars indicate mean. 1-way ANOVA on log-transformed values, with Tukey post-test. **g**, Relative expression of proteins on respective populations of primary transferred (Input) and retransferred (Output) cells 28 d post-tumour inoculation determined by flow cytometry with unbiased hierarchical clustering indicated and **h**, PCA indicating expression of proteins in (**g**), each dot being concatenated samples from independent experiments. **i**, related to Fig. 6e–i, showing total cell counts isolated from tumours at d 22 post-tumour inoculation. Statistics: **a**, pooled from 3 independent experiments, with 10 total replicates (that is M1-10). **b**, M1 and M2 indicate independent experiments. **d-h**, Data pooled from 3 independent experiments, N=11 (T_PROG_), 23 (T_EX_), 17 (T_RM_) mice. **i**, Data representative of 2-independent experiments, linked symbols indicate cells isolated from the same mouse, N=9 mice per group, analysed by 2-way repeated-measures ANOVA with Sidak post-test. Panel **d** adapted from ref. ^47^, Springer Nature America.

Minimal barcode sharing was observed between mice receiving 2 × 10^3^ OT-I T cells, and sharing was detectable only in mice that received 1 × 10^4^ cells (Extended Data Fig. 9a), indicating that most transferred barcodes were unique, minimizing the possibility of PCR artifacts. We excluded any barcodes identified across several mice before assessing barcode sharing across splenic and tumor populations in individual mice. These analyses showed that antigen-specific, tumor-derived T_PROG_, T_EX_-like and T_RM_ cells were more similar to each other, and distinct from CD69^−^ and spleen-derived populations (Fig. 6b,c). We conducted a parallel experiment using T_EFF_ OT-I cells transduced with SPLINTR-encoding retrovirus, transferred into AT3-OVA-bearing mice. Barcode diversity was again determined across spleen and tumor populations, confirming that barcoded OT-I library pools were unique (Extended Data Fig. 9b). As per the naive T cell experiment, barcode distribution reinforced that tumor-derived T_PROG_, T_EX_-like and T_RM_ cells displayed a high degree of barcode overlap and were distinct from CD69^−^ and spleen-derived populations (Fig. 6d and Extended Data Fig. 9c). Altogether, these data indicate that tumor-specific T_RM_ cells do not arise from a distinct T cell lineage but instead share common progenitors with other intratumoral populations.

The developmental association between each cell type left unresolved whether tumor-antigen-specific T_RM_ cells that develop after antigen loss or following spatial segregation from cognate antigen can transition into T_EX_ cells upon antigen re-encounter. To investigate this, we isolated intratumoral OT-I T cells exhibiting T_PROG_, T_EX_-like or T_RM_ phenotypes from AT3-OVA tumors and retransferred them intravenously (i.v.) into secondary recipient mice bearing AT3-OVA tumors (Extended Data Fig. 9d). Among these populations, T_EX_-like OT-I T cells were significantly less efficient at repopulating tumors compared to T_PROG_ or T_RM_ cells (Extended Data Fig. 9e,f). Due to the relatively low recovery of transferred cells, we performed composite protein expression profiling from each transferred group by experiment, and compared these profiles to T_PROG_, T_EX_-like and T_RM_ OT-I T cells isolated from concurrently analyzed primary tumors (Extended Data Fig. 9g,h). This analysis revealed that all retransferred populations in secondary tumors most closely resembled the T_EX_-like phenotype observed in primary tumors.

To determine whether differences in trafficking influenced the ability of cells to repopulate tumors and adopt distinct phenotypes, we sorted congenically distinct T_EX_-like and T_RM_ OT-I T cells from primary AT3-OVA tumors and cotransferred them directly into secondary tumors that either expressed or lacked OVA (Fig. 6e). Notably, direct intratumoral transfer eliminated the repopulation advantage observed previously for T_RM_ cells, potentially reflecting their enhanced ability to repopulate distal sites following i.v. transfer (Extended Data Fig. 9i). In the absence of cognate antigen, transferred T_RM_ cells largely retained their phenotype, whereas approximately 50% of the heterogenous T_EX_-like population (input, comprising TOX^Lo^ and TOX^Hi^) could adopt the T_RM_ phenotype (Fig. 6f,g). The dysfunctional TOX^Hi^ population was absent within AT3 tumors lacking antigen, consistent with data showing that these cells are sustained in the presence of antigen (Fig. 6f,g). Conversely, when the same populations were transferred into AT3-OVA tumors, a fraction of T_RM_ cells maintained their phenotype; however, most transitioned to a T_EX_-like state, including a substantial proportion acquiring the TOX^Hi^ phenotype (Fig. 6h,i). Thus, T_RM_ cells can be driven towards terminal exhaustion within the antigen-rich environment of secondary tumors. Overall, these data indicate that, unlike nontumor reactive bystander T_RM_ cells, tumor-specific T_RM_ and T_EX_ populations can arise from a common origin, with tumor-specific T_RM_ cells having the capacity to be driven towards exhaustion upon chronic antigen re-encounter.

## Discussion

Our study reconciles two critical T cell subsets associated with tumor control, namely CD8^+^ T_RM_ and T_EX_ cells. We show that these subsets are distinct T cell populations that have been conflated in the literature. This has arisen due to T_EX_ cells engaging a residency program that relies on the same transcriptional machinery used by T_RM_ cells to inhibit tissue egress. Thus, T_RM_ gene signatures developed without T_EX_ cell consideration cannot disentangle these two populations. This study resolves this issue by establishing broad gene signatures that reliably distinguish these subsets across human tumors. Our approach to disentangle T_RM_ and T_EX_ cells in tumors provides a framework for identifying other phenotypically and transcriptionally similar populations. We also emphasize that, just as T_RM_ cells exhibit tissue-specific phenotypes^47–49^, the features of tumor-associated T_RM_ and T_EX_ cells also vary between tumor types. Accordingly, the use of specific markers and gene signatures should be validated in the relevant biological context.

The deconvolution of tumor-associated T_RM_ and T_EX_ cells highlights their potential to play distinct roles in anti-tumor immunity and their differential impact on ICB responses. High T_EX_ gene signature expression in BC correlates with positive ICB outcomes consistent with the elevated expression of PD-1 and CTLA4 by T_EX_ cells. The association of high TMB with increased T_EX_ cell frequency suggests that increased new tumor epitopes enable T_EX_ cell development. T_EX_ cells, as defined in this study, consistently co-express CD39 and CD103—markers known to enrich for tumor-specific CD8^+^ T cells^44,45,50^. High TMB is generally associated with better ICB responses, including BC subtypes such as TNBC, where TMB predicts favorable outcomes to therapies such as pembrolizumab^51–54^. The observed link between high T_EX_ gene signature scores, TMB and ICB efficacy underscores the critical role of T_EX_ cells in tumor control.

We found that most tumor-associated T_RM_ cells identified in humans are clonally and developmentally distinct from T_EX_ cells. Tumor-antigen agnostic T cells, including T cells specific for previous infections, can adopt a residency phenotype within tumors. Consistently, we show that TCR signaling is antithetical to bona fide T_RM_ cell formation in tumors, aligning with the conceptual notion that immunological memory can develop only following clearance of cognate antigen. However, we also reveal that tumor-antigen-specific T_RM_ cells can develop in settings of reduced TCR signaling and diminished antigen sensing. Tumor-specific T_RM_ cells probably exist in lower numbers compared to tumor-specific T_EX_ cells as it is expected that they have encountered less antigen, and therefore have had reduced proliferative bursts, accounting for the limited clonal sharing that we observe between T_RM_ and T_EX_ cells in human tumors.

Although T_RM_ gene signatures are associated with improved overall survival in BC patients, the mechanisms underlying T_RM_-mediated tumor control remain unclear. The accumulation of T_RM_ cells may coincide with other features of tumor control, such as tumor antigen clearance. However, our analyses suggest that tumor-specific T_RM_ cells persist in healthy tissues adjacent to tumors, raising the possibility that they contribute to long-term immune surveillance and protection against tumor recurrence. The clinical benefit of tumor-specific T_RM_ cells may lie in their ability to maintain equilibrium with residual cancerous cells, preventing tumor recurrence, or in prophylactic settings following vaccination^55^. Given that these cells form following antigen clearance and can re-enter tissues and repopulate distal sites, the presence of T_RM_ cells in human tumors may reflect an effective immune response capable of providing durable protection at both primary and metastatic sites.

Finally, substantial populations of bona fide, nonexhausted, tumor-agnostic T_RM_ cells can be identified in diverse tumors. These cells maintain superior functionality in humans and murine models but are not targeted by current immunotherapies. Thus, approaches to activate bystander T_RM_ cells through TCR-independent pathways or administration of viral peptides^45,56,57^, or to mobilize functional tumor-specific T_RM_ cells, combined with current T_EX_ cell-targeted ICB therapies could raise the ceiling of effective anti-tumor responses.

## Methods

### Mice

C57BL/6, gBT-I:CD45.1.2, gBT-I:CD45.1.1, gBT-I:CD90.1.2, P14:CD45.1.2, OT-I:CD45.1.2, OT-I:CD90.1.2; OT-3:CD45.1.2:TCRα^−/−^ (ref. ^59^), OT-I:TGFβRII^fl/fl^:Cre^dLck^:CD45.1(TGFβRII^−/−^) and TCRα^fl/fl^Cre^ERT2^ female mice were bred and maintained in the Department of Microbiology and Immunology, University of Melbourne under a 12 h/12 h light/dark cycle, at 19–22 °C and 40–70% humidity. All mice were maintained on standard chow. All experiments were approved by the University of Melbourne Animal Ethics Committee (ID nos. 21651 and 21938). All mice were between 6 and 14 weeks of age at the beginning of the experiments.

### Isolation of lymphocytes from human tumors and tissues

Following tumor excision, a representative tumor fragment was processed to generate single-cell suspensions as described previously^1^. Briefly, tumor or healthy tissues were diced finely in RPMI1640 containing 10% FCS and 0.5 mg ml^−1^ collagenase D (Worthington Biochemical) and were incubated for 30 min at 37 °C. Digested pieces were mashed through 70-μm strainers and washed with RPMI1640 with 10% FCS. Lymphocytes from liver tumors and healthy liver tissues were enriched through density gradient centrifugation (500*g*, 20 min at 25 °C) on a 44%/70% isotonic Percoll gradient (GE Healthcare), diluted in HBSS, and cells at the solution interphase were isolated and washed with HBSS. Blood was diluted 1:1 in HBSS, overlaid on Ficoll-Paque PLUS (Sigma-Aldrich), centrifuged (400g, 15 min, 25 °C), and peripheral blood mononuclear cells were isolated from interphase. Cells were either used immediately for flow cytometry, or frozen in 10% dimethylsulfoxide:90% FCS freezing medium (breast, breast tumor, and blood samples) or Cryostor CS10 Freeze Medium (Stem Cell Technologies, cat. no. 07930; liver and liver tumor samples) for CITE-seq and restimulation assays.

### Mouse tumor and infection models

The TNBC cell line AT3-OVA was provided by P. Darcy (Peter MacCallum Cancer Center)^60,61^. AT3-OVA cells were cultured with complete DMEM (DMEM, 10% FCS, 2 mM L-glutamine, 100 U ml^−1^ penicillin, 100 mg ml^−1^ streptomycin). A total of 5 × 10^5^ AT3-OVA cells in exponential growth phase (~70–80% confluency) were injected orthotopically into the fourth mammary fat pad in a total volume of 50 µl HBSS.

The melanoma B16F1-gB.GFP (B16-gB) cell line was provided by J. Waithman (University of Western Australia)^55^. B16-gB cells were cultured and passaged in complete RPMI (RPMI1640, 10% FCS, 2 mM L-glutamine, 100 U ml^−1^ penicillin, 100 mg ml^−1^ streptomycin, 50 mM 2-mercaptoethanol) at 37 °C and 5% CO_2_. B16-gB was inoculated epicutaneously as described previously^55^. Tumors were measured every 2–3 days following the development of palpable tumors using vernier callipers, and tumor volume was calculated (length × width^2^)/2. Mice were euthanized when tumors reached an ethical limit of 1.0 cm^3^. gBT-I or OT-I T_EFF_ cells were transferred into mice following the observation of tumor growth, between 14 and 20 days following tumor inoculation, and then collected at tumor endpoint (when tumors reached a maximum of 1,000 mm^3^), which occurred 14–20 days following T cell transfer. AT3-OVA tumors were measured similarly and mice were euthanized before tumors reached the ethical endpoint volume of 1,000 mm^3^.

LCMV infection was performed by intraperitoneal (i.p.) injection of 2 × 10^5^ pfu of the Armstrong strain of LCMV.

### T cell transfer

For the adoptive transfer of naive T cells, 1–5 × 10^4^ transgenic (P14 or OT-I) T cells were transferred i.v. to recipient mice 1–2 days before infection with LCMV or inoculation with AT3-OVA. For effector T cell transfer, transgenic (P14, OT-I, OT-3 or gBT-I) T cells were activated in culture for 5 days with gp_33-41_ (KAVYNFATM–P14; Auspep), OVA_257-264_ (SIINFEKL–OT-I/OT-3; Auspep), or gB_498-505_ (SSIEFARL–gBT-I; Auspep) peptide-pulsed splenocytes, in the presence of recombinant human IL-2 (25 U ml^−1^; Peprotech) in complete RPMI (as above) at 37 °C and 5% CO_2_. T cells were split 1:1 with fresh medium and IL-2 on days 2–4. T cells were resuspended in 200 µl HBSS for IV transfers. Unless stated otherwise, 1 × 10^4^ effector OT-I, 5 × 10^6^ gBT-I or 1 × 10^6^ OT-3 were transferred 10 days post-AT3-OVA inoculation.

### In vivo treatments

For in vivo intravascular staining, 3 μg of αCD90.2-biotin was injected i.v. in 200 μl PBS, 3 min before euthanasia. To inhibit S1P-signaling pathways, mice were administered FTY720 (Cayman Chemical) diluted in 2% (2-hydroxypropyl)-β-cyclodextrin (Sigma-Aldrich) or vehicle daily via i.p. injection (1 μg g^−1^) for the times indicated in the figure legend (Extended Data Fig. 7c). For tamoxifen treatment, mice were administered 2 mg of tamoxifen (or ethanol as vehicle control) diluted in sunflower seed oil (both from Sigma) i.p. daily for a total of five injections.

### Isolation of lymphocytes from mouse tissues

Lymphocytes from spleens and lymph nodes were isolated by grinding through 70-μm strainers. AT3-OVA tumors and MFPs (avoiding the inguinal lymph node) were collected into collagenase III solution (3 mg ml^−1^; Worthington) containing DNase I (2.5 mg ml^−1^; Sigma), chopped into fine pieces and incubated for 60 min at 37 °C. B16-gB tumors were collected into liberase TL research grade solution (0.25 mg ml^−1^; Roche), chopped into fine pieces, and incubated for 60 min at 37 °C. Spleens and tumors were passed through a 70-mm strainer and erythrocytes were lysed in red cell lysis buffer (eBioscience) before staining for flow cytometry. Cells isolated from B16-gB tumors were cryopreserved in 10% dimethylsulfoxide:90% FCS for scRNA-seq.

### Flow cytometry and cell sorting

Single-cell suspensions were stained with fluorescently conjugated antibodies at 4 °C for 30–45 min in FACS buffer (1% BSA and 0.05 M EDTA in PBS). Dead cells were excluded by staining with Zombie Aqua or Near Infrared dyes (Biolegend). For transcription factor and cytokine staining, samples were fixed and permeabilized using the Foxp3/transcription-factor-staining buffer kit (eBioscience) according to the manufacturer’s instructions and stained with fluorescent antibodies against intracellular proteins in permeabilization buffer (containing 2% rat and mouse serum (eBioscience)). All relevant information regarding the antibodies used in this study (clone, manufacturer, catalog number and dilution) is provided in Supplementary Table 1. Cells were enumerated using SPHERO calibration particles (BD Biosciences). Fluorescently labeled cells were acquired on a Cytek Aurora, unmixed with SpectroFlo software and analysis was performed using FlowJo (v.10.10.0; Treestar), or OMIQ for high-dimensional flow cytometry analysis. For cell sorting experiments, cells were sorted using a BD FACS Aria, using a 100-μm nozzle. For human cell sorting experiments for CITE-seq, CD3^+^ Zombie NIR^−^, cells were sorted into 50% FCS in RPMI before downstream processing. For B16-gB sorting experiments, DAPI^−^ OT-I or gBT-I cells were sorted into 50% FCS in RPMI before downstream processing. For mouse AT3-OVA sort-transfer experiments, mice were treated with anti-ARTC2 nanobody i.v. (50 mg per mouse; S+16a, Biolegend) 10 min before organ collection, stained as above, and respective populations (see figure legends) sorted into 50% FCS in RPMI before washing with HBSS and transfer into recipient mice (8 × 10^4^ cells in 200 µl i.v.).

### In vitro stimulation assays

To assess cytokine production capacity by T cells, cells were stained with surface stain antibodies (as above) and then incubated with phorbol myristate acetate (PMA; 50 ng ml^−1^; Sigma-Aldrich), Ionomycin (1 mg ml^−1^), Brefeldin A (10 mg ml^−1^, Sigma-Aldrich), GolgiStop (1:1,500, BD) in complete RPMI (as above) for 4–5 h before intracellular staining and flow cytometry. Unstimulated controls were included to confirm that stimulation did not alter cell-surface staining and to serve as negative controls for staining.

### Bulk RNA-seq analysis

Scatter plot of log_2_ fold changes (FCs) (for Fig. 1a) was produced using raw RNA-seq count data from GEO for Man et al.^26^ (accession GSE84820) and Mackay et al.^2^ (accession GSE70813). From the Man data, wild-type day 30 chronic and acute LCMV samples were selected for analysis, whereas, from the Mackay data, the following samples were selected for three separate analyses: (1) Skin T_RM_, T_CM_ and T_EM_ HSV samples; (2) Gut T_RM_, T_CM_ and T_EM_ LCMV samples; and (3) Liver T_RM_, T_CM_ and T_EM_ LCMV samples. T_CM_ and T_EM_ samples were subsequently treated as a single ‘T_CIRC_’ group. For each analysis, genes were annotated with information from the National Center for Biotechnology Information and those with obsolete symbols or annotated as rRNA were removed, as were genes that failed to achieve a count above ten in all samples in at least one group. Each dataset was further processed by applying the imputation strategy published previously^47,62^. Counts-per-million values were calculated using the edgeR package^63^, together with scaling factors derived from the TMM method^64^, log_2_ transformed with a previous count of 1, followed by application of the normalization method RUV-III^65^ with biological replicates nominated as replicates, mouse housekeeping genes^56^ nominated as ‘negative control’ genes, and *k* = 1 factors of unwanted variation. Normalization success was assessed with relative log expression plots^66^, PCA plots^67^, and histograms of *P* values. The limma package^68^ was used to fit gene-wise linear models for the given group structure with the output from RUV-III as an additional model covariate. The log_2_FC estimates from the Mackay Skin, Gut, and Liver analyses were averaged to create a ‘pooled T_RM_’ expression profile and then plotted against the estimates from the Man analysis. A *P* value was calculated by constructing a two-way contingency table, counting the number of genes with concordant/discordant log_2_FCs, and applying Fisher’s exact test for association. Core T_RM_ gene signature was used as described^69^.

### CITE-seq and scRNA-seq library preparation

Following sorting of cell populations, sorted cells were stained with TotalSeq-C Universal Cocktail v.1.0 (Biolegend; Human CITE-seq experiments) or TotalSeq-C Hashtag antibodies (Biolegend; mouse B16-gB scRNA-seq experiment) according to the manufacturer’s instructions. Cells were filtered through a 40-mm Flowmi cell strainer, loaded onto a 10x Genomics chromium controller, and prepared for sequencing using a Chromium Next GEM Single Cell 5′ kit with feature barcoding and immune receptor mapping (v.2, Dual Index) and VDJ enrichment kits for mouse or human TCR from 10x Genomics. Libraries were generated according to the manufacturer’s instructions. Libraries were profiled on an Agilent Tapestation and quantified using a Qubit before sequencing on an Illumina NextSeq2000 P2 or P3 kit.

### CITE-seq and scRNA-seq analysis

Sequencing reads of three separate lanes were aligned to the hg38 reference genome and TCR reference (VDJ) and counted with cellranger v.6.1.2. CITE-seq antibodies were aligned to their custom reference sequences from BioLegend. Patient samples were demultiplexed into genotypic donors using vireo^70^ on aligned BAM files from three lanes. Donors were matched to genotypes using known cell frequencies in each sample. Three batches of single-cell data were merged and processed using Seurat^71^ (v.4.3.0). Specifically, cells were filtered if they contained fewer than 500 genes, more than 5% mitochondrial RNA and were annotated to have more than one beta chain in the VDJ assay or two genotypes in the vireo analysis (cell doublets). Then, for the RNA assay, we used NormalizeData to normalize the counts data and determine the top 2,000 variable genes using FindVariableFeatures. We excluded TCR components (^TRA/^TRB), mitochondrial genes (^MT), and ^HLA from the variable genes as unwanted factors of variance and performed PCA (RunPCA). The CITE-seq assay was processed similarly with centered log ratio normalization and margin = 2. We removed the individual effects between the donors using Harmony^72^ on the RNA-seq and CITE-seq assays individually and then combining the correction reductions using the FindMultiModalNeighbors functions (using 25 dimensions from the RNA reduction and 18 from CITE-seq). Uniform manifold approximation and projection (UMAP) reductions and cell neighbors were calculated using 25 dimensions from the weighted nearest-neighbor reduction. Clusters were detected with a resolution of 3. We then determined T cell subsets within the data by assessing cluster markers (FindAllMarkers) and their overall expression and comparing them to published markers in the literature (merging clusters as need be). We subset the data to CD8^+^ T cells only at this point by excluding other T cell populations and contaminating immune populations. All further analyses and plots were generated in R (v.4.2) using tidyverse^73^ functions and ShinyCell^74^. Heatmaps were created with the pheatmap package (v.1.0.12). Subset signature expression levels were calculated using the AddModuleScore function. Pseudobulk analysis was performed using the edgeR package^63^: counts of reads were summed per subset. Pseudobulk samples from the periphery of the data (gdT, MAIT) are filtered from the analysis and lowly expressed genes removed using filterByExpr(). The samples were then normalized using TMM and the calcNormFactors() function. Dimensional reduction was performed using the plotMDS function. Other single-cell datasets, such as Giles 2022^27^, or subsets of the data (tumor only) were analyzed analogously to the methods above (leaving out CITE-seq and TCR where appropriate).

#### TCR analysis

TCRs from cellranger outputs were paired based on cell barcodes and merged with gene expression data. In cases where several contigs were detected the contigs with the highest unique molecular identifier was kept. TCR clonotype was defined by the joined alpha and beta CDR3 nucleotide sequences, and expanded TCR clonotypes were determined by filtering the list of clonotypes to those that are found in two or more cells. Antigen specificity of cells was based on TCR clonotype and donor HLA identity. HLA type was determined with ArcasHLA using the ‘—single’ flag^75^. Reads covering the genome coordinates chr.6:28510120–33480577 were extracted into a separate fastq file per donor and processed individually. Plots of clonotype diversity and similarity within/across cell types were calculated using djvdj (https://rnabioco.github.io/djvdj/ v.0.1.0) and plotted with ComplexHeatmap^76^. For clonotype sharing plots, expanded clonotypes within each tissue were filtered to include only those that are shared across more than one subset. The number of shared clonotypes was quantified for each subset pairing, and the total number of cells contributing to clonotype sharing from each subset was counted. Visualizations were generated with custom R scripts using the ggraph package (v.2.0.5). For iterations of the sharing plots with filtering, input data were refined so that a shared TCR was removed from the plot if one of the cell types in the pairing contributed just one cell (for *n* > 1 filtering) or two or fewer cells (for *n* > 2 filtering). Other visualizations of clonotype cell fate and cluster sharing were generated using custom R scripts, as provided. Comparisons between TCR sequences was performed using TCRdist3 (refs. ^39,40^) using default weights with the distance matrix calculated as the sum pairwise distance for the alpha and beta chains. To determine potential virus-recognizing clones, published TCRs with known viral HLA-peptide specificities were obtained from VDJdb^41–43^, following which edit distances were calculated for each clone against reference CDR3a/b peptide sequences. The lowest edit distance was obtained for each clone, with distances less than 1 considered viral-associated.

#### Pancancer atlas processing

Preprocessed Seurat objects were obtained^28^. SCTransform was used to normalize, scale and regress prefiltered dataset. Precomputed scores for dissociation induced genes(DIG), Malat1, mitochondrial percentage and cell-cycle(G2M and S scores) were used for regression. T cell receptor variable/joining (^TR[A|B|G|D][V|J]), B cell receptor variable/joining (^IG[H|L|K][V|J|D]) DIG(^HSP/^DNAR), cell-cycle, DIG(^HSP/^DNAJ) and ribosomal (^RP([0-9]^+^-|[LS])) genes were removed from VariableFeatures gene list before calculating PCs. Subsequent UMAP and FindNeighbors commands performed with 15 PCs. T_RM_ and T_EX_ scores were calculated with the AddModuleScore command using respective genesets. T_EM_ and T_EMRA_ scores were calculated using differentially expressed genes calculated from the BC dataset. Other external datasets were analyzed similarly.

### Signature acquisition and module scoring

T_RM_ and T_EX_ expression signatures were derived by summing raw counts, gene-wise, over each donor within each cluster, to create ‘pseudobulk’ samples for each donor/cluster combination. Samples composed of fewer than 20 cells were removed, and samples from the same metacluster were subsequently treated as belonging to the same group. Genes annotated as being of ‘HLA’, ‘TRB’, ‘TRA’, ‘TRG’ or ‘TRD’ type were removed, as were genes that failed to achieve a count above 5 in at least two samples in at least one group (breast) or a count above 3 in at least four samples in at least one group (liver). Counts were log_2_ transformed with a previous count of 1/2 (breast) or 1 (liver), followed by application of the normalization method RUV-III with samples from the same group nominated as replicates, human housekeeping genes nominated as ‘negative control’ genes, and *k* = 20 (breast) or *k* = 15 (liver) factors of unwanted variation. Normalization success was assessed as above. The edgeR package [*] was used fit gene-wise negative binomial generalized linear models for the given group structure with the output from RUV-III as additional model covariates, with a previous count of 1/2 (breast) or 1 (liver). Likelihood ratio tests were employed to test for DE, where a gene was judged to be DE if the Benjamini and Hochberg^77^ adjusted *P* value < 0.05. For breast, the T_RM_ signature was defined by a contrast between the T_RM_ group and the average of all other groups; the T_EX_ signature was defined similarly. For liver, the T_RM_ signature was defined by a contrast between the CD103^+^ T_RM_ group and the average of all other groups except the CD103^−^ T_RM_ group; the T_EX_ signature was defined similarly. The breast T_RM_ ‘union’ signature was derived using the same steps for breast above, except that samples obtained from the T_RM_ or T_EX_ clusters were combined into a single group. Signature scores were calculated from the SCTransformed assay using the ‘AddModuleScore’ function from the Seurat package^71^ for up and down genes separately and combined by averaging the scores for the up genes and the sign reversed scores for the down genes. These scores were overlaid onto UMAP plots using the FeaturePlot function from Seurat. To generate the pancancer T_RM_ signature we focused on the leading-edge genes contributing to the enrichment in the barcode plots (Fig. 3c and Extended Data Fig. 4g). Specifically, the leading-edge genes contributing to T_RM_ signature associations between the BC and liver datasets were refined by intersecting the genes in the breast and liver T_RM_ signatures (described above) and then subsetting to genes which are either (1) concordantly DE in both breast and liver or (2) DE in one with a concordant absolute log_2_FC > 0.5 in the other. The pancancer T_EX_ signature was refined similarly (Extended Data Fig. 4g). Barcode enrichment plots were generated using limma, and gene set enrichment *P* values were calculated using the camera function on the log_2_FC values for populations of interest against the background calculated with FindMarkers. All signatures are available in Supplementary Table 2.

### Survival analysis

Clinical information and normalized microarray data (for Fig. 2l and Extended Data Fig. 3f–h) for the study^58,78^ were downloaded from the cBioPortal (https://www.cbioportal.org). Signature scores for each patient were calculated using the ‘sig.score’ function from the genefu package^79^. Kaplan–Meier curves were calculated using the R package survival and were plotted, with the log-rank test *P* value, using the R package survminer.

Clinical information and microarray data (for Fig. 2m) for the iSPY study^31^ were downloaded from supplementary material and GEO (accession GSE194040), respectively. Probes targeting the same gene were averaged. If a gene had missing values, these were imputed using the average of all nonmissing values across the gene. For each platform batch, relative log expression^66^ values were computed, the median value for each sample was calculated, and then samples were ranked by this median value: the bottom, middle and top third rankings defined three separate ‘pseudo-batches’ of samples. For each PAM50 BC expression subtype in each pseudobatch, one of the following was performed: if there were between five and ten samples, five samples were selected randomly and averaged, gene-wise, to create one ‘pseudo-sample’; or if there were more than ten samples, the bottom five ranked samples and the top five ranked samples were averaged, gene-wise, to create two pseudosamples. These pseudosamples were used as pseudoreplicates in the RUV-III-PRPS normalization methodology^80^, with all genes nominated as ‘negative control’ genes, and *k* = 7 factors of unwanted variation. Normalization success was assessed with relative log expression and PCA plots^67^. Based on this normalized data, signature scores for each patient were calculated using the ‘sig.score’ function from the genefu package. Receiver operating characteristic (ROC) curves and *P* values were calculated using the R packages pROC^81^ and verification, respectively.

### CycIF of human breast tumors

FFPE-embedded tumor tissues from seven patients (six female, one male) were purchased from the Cooperative Human Tissue Network based on histologic criteria (Supplementary Table 3), for invasive carcinoma (ductal/lobular) and 5-µm thick sections were cut on Superfrost Plus histology slides (Fisher scientific) at the BWH Histopathology core, as described previously^29^.

FFPE tissues were deparaffinized, rehydrated, subjected to antigen retrieval on a Leica BondRx and characterized by CycIF imaging, as described previously^29^. Briefly, tissues were stained overnight at 4 °C with primary antibodies or for 1 h at room temperature for secondary antibody conjugates in a dark, humidified chamber with antibodies diluted in Superblock (Thermo-Fisher) supplemented with 1 mg ml^−1^ Hoechst 33258 (Bio-Rad Laboratory) rinsed for 30 min in PBS (room temperature) and mounted with 50% glycerol in PBS using a 24 × 60-mm coverslip. All samples were stained and imaged in together to reduce batch effects using the antibody panel (Supplementary Table 1). Stained tissues were imaged using a Cytefinder II HT (Rarecyte) automated slide scanning fluorescence microscope using a ×20 (0.6 numerical aperture) objective. After imaging, mounted slides were soaked in PBS (room temperature) to detach coverslips, and then immersed in PBS supplemented with 4.5% hydrogen peroxide and 24 mM sodium hydroxide and exposed to LED light for 1 h. Slides were then rinsed twice in PBS in preparation for the next staining cycle. Image processing, assembly, segmentation and single-cell quantification were performed using MCMICRO^30^.

CycIF Images were processed with Cecelia^82^. Individual channels were denoised and segmented with Cellpose^83^ using a radius of 10 μm and 8 μm, respectively. T cell (CD3e) and cancer cell (panCK) segmentation was based on their respective markers in combination with the DNA (Hoechst-stained nuclei) channel from the third imaging cycle, the same cycle as CD3e. Cell populations were gated using Cecelia’s histocytometry module and spatial interactions analyzed. Cell distances were extracted using K-nearest-neighbor from the DBSCAN library^84^. Clusters within the CD8^+^CD103^+^KLF2^−^ T cells were defined using the Seurat package. We first removed the individual donor effects and performed PCA using a set of 22 features (CD39, CD45RO, CD3E, CD25, CD73, CD8A, GZMB, CD103, LAG3, PD-1, TIM3, CD69, TCF1, FOXP3, GNLY, CD4, pJUN, pERK, KLF2, CD7, CD94, NKG2A) (RunPCA). Shared neighbor graphs (FindNeighbors) and UMAP reduction (RunUMAP) were performed using the first ten dimensions. Clusters were detected using a resolution of 0.8 (FindClusters).

### CRISPR–Cas9 editing of CD8^+^ T cells

CRISPR–Cas9 editing of naive CD8^+^ T cells was performed as described previously^85^. Single-guide RNAs (sgRNA) targeting: *Tcf7* (5′-UCUGCUCAUGCCCUACCCAC-3′, 5′-AGCUGGGGGACGCCAUGUGG-3′, 5′-UGUGCACUCAGCAAUGACCU-3′), *Cxcr3* (5′-GAACAUCGGCUACAGCCAGG-3′, 5′-UGAGGGCUACACGUACCCGG-3′), *Cxcr6* (5′-UCUGUACGAUGGGCACUACG-3′, 5′-UGUGCCAAAGACCCACUCAU-3′) and *Cd19* (5′-AAUGUCUCAGACCAUAUGGG-3′, 5′-GAGAAGCUGGCUUGGUAUCG-3′) were purchased from Synthego (CRISPRevolution sgRNA EZ Kit). sgRNA/Cas9 RNPs were formed by incubating 0.3 nmol of each sgRNA with 0.6 μl Alt-R S.p. Cas9 nuclease V3 (10 mg ml^−1^; Integrated DNA Technologies) for 10 min at room temperature. Naive CD8^+^ T cells were negatively enriched from spleen and lymph nodes of gBT-I or OT-I mice by incubating cell suspensions with anti-CD4, anti-CD11b anti-F4/80, anti-Ter119 and anti-I-A/I-E monoclonal antibodies, followed by incubation with goat anti-rat IgG-coupled magnetic beads (Qiagen) before removing bead-bound cells. A total of 1 × 10^7^ enriched T cells were resuspended in 20 μl of P3 (P3 Primary Cell 4D-Nucleofector X Kit; Lonza), mixed with sgRNA/Cas9 RNP and electroporated using a Lonza 4D-Nucleofector system (DN100). Cells were rested for 30 min in a 96-well plate before direct transfer into recipient mice (cotransfer of 1:1 ratio of sg*Cd19*:sg*Tcf7* CRISPR-edited naive gBT-I cells, total of 2 × 10^4^ cells per mouse: Extended Data Fig. 8a–d) or activation in culture for 5 days with peptide-pulsed splenocytes (gB_498-505_ (SSIEFARL)) in the presence of IL-2 (25 U ml^−1^, Peprotech) at 37 °C, 5% CO_2_, before transfer into recipient mice (cotransfer of 1:1 ratio of sg*Cd19*:sg*Cxcr3 or* sg*Cd19*:sg*Cxcr6* CRISPR-edited naive gBT-I cells, total of 1 × 10^6^ cells per mouse; Extended Data Fig. 8m,n).

### SPLINTR methods

To construct the SPLINTR barcoding systems, violet-light excited GFP (VEX) was used to replace NGFR in MSCV-IRES-NGFR^86^ using the *Nco*I and *Bam*H1 restriction sites, or eGFP into the SPLINTR lentivirus vector^46^. A semi-random oligonucleotide library synthesized by Integrated DNA Technologies with the following pattern (NNSWSNNWSW)_6_ was amplified by eight cycles of PCR. The barcode library was cloned into the 3′ UTR of VEX using *Bam*H1 and *Mfe*I restriction sites at a 50:1 insert:vector ratio. Ten side-by-side ligation reactions were pooled and purified using two Monarch PCR and DNA Clean up columns (NEB) in a volume of 6 µl per column. The ligation reactions were pooled and split across two 25-µl aliquots of Endura Electrocompetent cells (Lucigen). Cells were recovered for 1 h postelectroporation, pooled and grown in 500 ml of LB supplemented with ampicillin (100 µg ml^−1^) overnight at 37 °C. The plasmid library was extracted using NucleoBond Xtra kit (Macherey–Nagel). SPLINTR libraries were sequenced to a depth of 100 million paired-end reads per technical duplicate and reference libraries used for downstream analysis were generated as described previously^46^. SPLINTR retrovirus and lentivirus VEX library represents a highly diverse barcode library containing 3.2 × 10^6^ unique barcodes or 1.3 × 10^6^ barcodes, respectively.

Naive (T_N_) OT-I T cells were barcoded via transduction of hematopoietic stem and progenitor cells (HSPC) and intrathymic transfer into sublethally irradiated chimeric mice. Briefly, bone marrow was isolated from OT-I mice, and lineage(lin)-positive cells were depleted using the EasySep Mouse Hematopoietic Progenitor Cell Isolation Kit (Stem Cell Technologies). Lin-negative HSPCs were plated in fibronectin-coated 12-well plates containing polyvinyl alcohol (PVA)-based medium: 1× Ham’s F-12 Nutrient Mix liquid media (Gibco) supplemented with 10 mM HEPES, 1× P/S/G, 1 ITSX, 1 mg ml^−1^ PVA along with TPO (100 ng µl^−1^) and mouse SCF (10 ng µl^−1^). For barcoding, 20 pools of 1 × 10^6^ cells (cultured for 6 days) were transferred to 24-well fibronectin-coated plates in 250 μl of PVA medium. Viral barcoding vectors were titrated to achieve 10% reporter expression (0.1 multiplicity of infection) to minimize multiple integrations. Cells were transduced via spinfection at 2,000*g* for 2 h (no break). After transduction, medium was refreshed and cells were cultured for a further 48 h before VEX-positive and Lin- cells were sorted (ARIA Fusion, BD). The 20 pools of 3.5–5.5 × 10^5^ cells were plated individually into 48-well plates and expanded for 1-week. Recipient C57Bl/6 mice were then irradiated 4 Gy and HSPCs were transplanted intrathymically such that each mouse received an independent barcode pool. Eight weeks later, chimeric mice were bled, equal numbers of VEX^+^ OT-I T cells from each mouse were pooled, and cells were transferred into C57Bl/6 mice that were then inoculated with AT3-OVA tumors.

Effector (T_EFF_) OT-I T cells were barcoded as follows. Naive OT-I T cells were isolated from spleens and LNs and enriched via negative selection as for CRISPR–Cas9 experiments. Enriched OT-I T cells were activated with anti-CD3 (2 μg ml^−1^, 145-2C11, BioXCell) and anti-CD28 (1 μg ml^−1^, 37.51, BioXCell) for 24 h before ‘spinfection’ with a pretitrated volume of SPLINTR retrovirus to ensure <5% (0.05 multiplicity of infection) transduction efficiency to limit several barcode integrations in a single-cell, in 24-well plates, precoated with Retronectin (32 μg ml^−1^, Takara). Transduced cells were sorted 24 h later and transferred into mice bearing AT3-OVA tumors. At the endpoint, transduced OT-I T cell populations were sorted from spleens and tumors and lysed in Viagen lysis buffer with 0.5 μg ml^−1^ proteinase K (Invitrogen) for DNA barcode sequencing.

Barcode sequences were amplified from genomic DNA using primers flanking the constant region of the barcode before adding i5 and i7 indexes compatible with next generation sequencing^46^. Libraries were sequenced on an Illumina NextSeq2000 using 100 bp single-end chemistry targeting 2 million reads per sample. The BARtab pipeline^87^ (https://github.com/DaneVass/BARtab) was used to map the sequencing reads to a barcode reference library, perform quality control analysis, and generate a barcode counts table. The bartools R package^87^ was used to collapse PCR replicates and generate barcode abundance bubble plots and correlation heatmaps for data visualization. Scatterplots comparing barcode abundance between two samples were generated using the R package ggplot2.

### Ethics approval

All animal experiments were approved by The University of Melbourne Animal Ethics Committee (ID nos. 21651 and 21938). All study on human specimens was approved by the Human Research Ethics Committee of the University of Melbourne (ID nos. 13009 and 14517). All participating patients provided written informed consent.

### Randomization and statistics

Test animals were assigned randomly to experimental groups in all experiments. No further randomization of data collection was performed. Data collection and analysis were not performed blinded to the conditions of the experiments. No statistical methods were used to predetermine sample sizes, but our sample sizes are similar to those reported in previous publications^25,47,62^. In all cases, data distribution was assumed to be normal, but this was not tested formally. No datapoints were excluded from the statistical analyses.

### Reporting summary

Further information on research design is available in the Nature Portfolio Reporting Summary linked to this article.

## Online content

Any methods, additional references, Nature Portfolio reporting summaries, source data, extended data, supplementary information, acknowledgements, peer review information; details of author contributions and competing interests; and statements of data and code availability are available at 10.1038/s41590-025-02347-9.

## Supplementary information

Supplementary Information Supplementary figure illustrating representative gating strategies utilized for flow cytometry and FACS analysis.

Reporting Summary

Peer Review File

Supplementary Table 1 List of antibodies used in the study.

Supplementary Table 2 Signature genes developed and utilized in the study.

Supplementary Table 3 Human donor information.

## Source data

Source Data Statistical source data for Figs. 1–6.

## Data Availability

The single-cell CITE-seq and scRNA-seq data generated for this study have been deposited in the GEO under accession codes GSE267552, GSE309625 and GSE309444. Source data are provided with this paper.

## Code availability

The code generated and used for analysis of single-cell CITE and RNA-seq data is provided via Github at https://github.com/CSI-Doherty/Burn2025.
