# Supplementary material for: Antigen reactivity defines tissue-resident memory and exhausted T cells in tumors
Source: Nat Immunol. 2025 Dec 29;27(1):98–109. doi: 10.1038/s41590-025-02347-9 (PMC12764432; doi:10.1038/s41590-025-02347-9)

nature immunology

Supplementary information https://doi.org/10.1038/s41590-025-02347-9

Antigen reactivity defines tissue-resident memory and exhausted T cells in tumors

In the format provided by the authors and unedited

Example gating strategy associated with human flow cytometry in Figure 1, Extended Data Figs. 1, 3

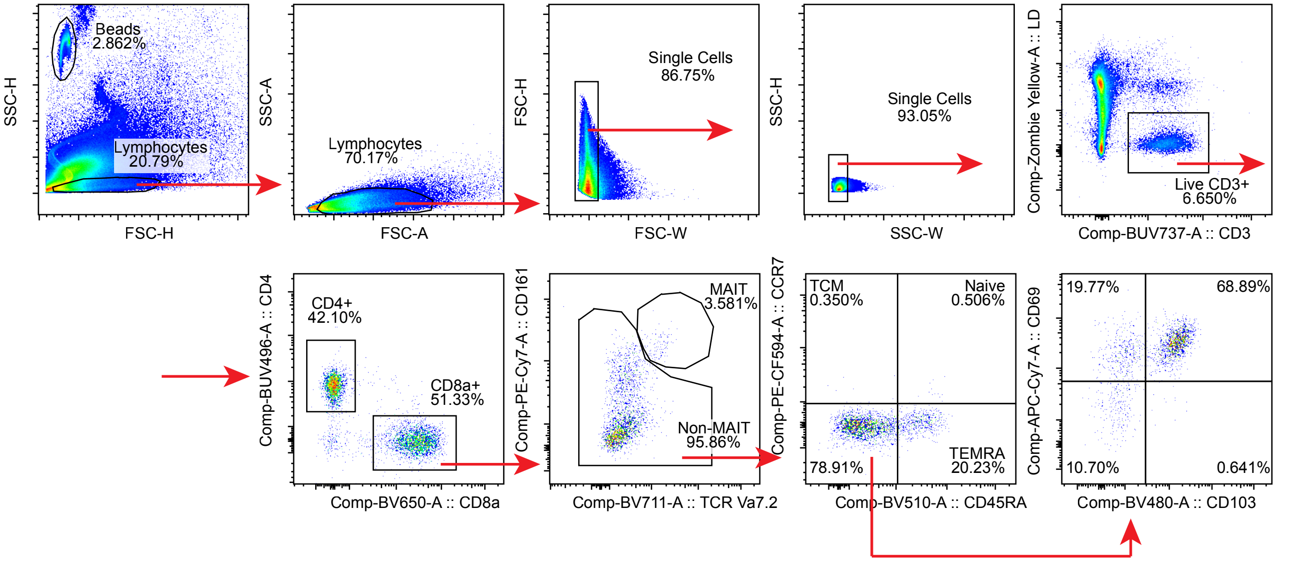

Example gating strategy associated with human flow cytometry in Figure 5,6; Extended Data Figs. 6-9

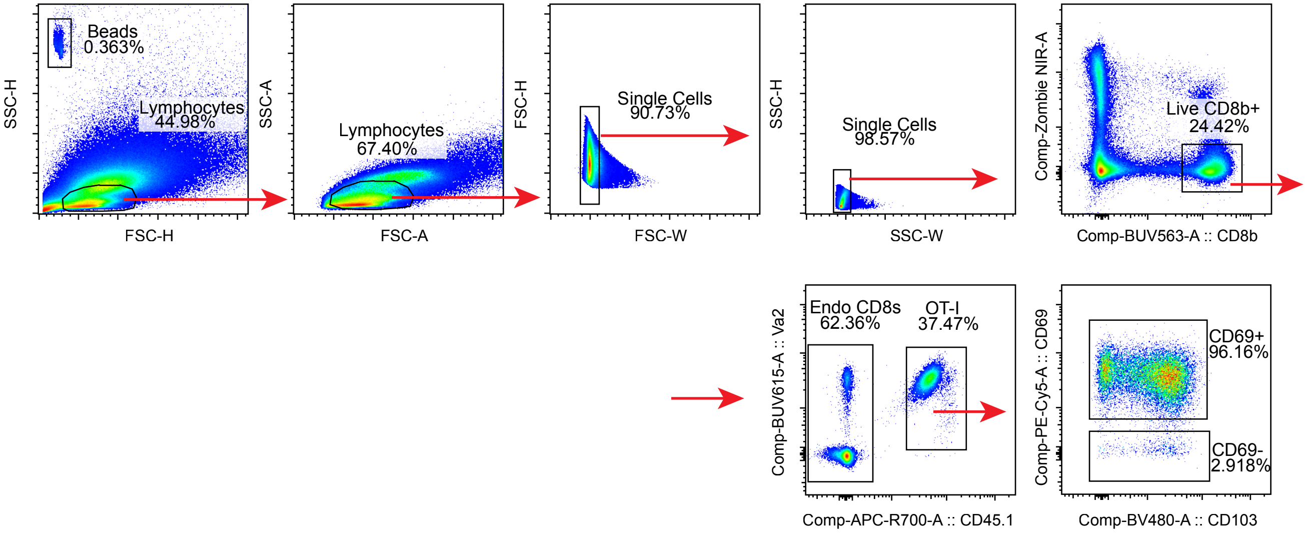

Supplement: Supplementary file 1 — Supplementary figure illustrating representative gating strategies utilized for flow cytometry and FACS analysis. [file 41590_2025_2347_MOESM1_ESM.pdf]
