# Supplementary material for: Antigen reactivity defines tissue-resident memory and exhausted T cells in tumors
Source: Nat Immunol. 2025 Dec 29;27(1):98–109. doi: 10.1038/s41590-025-02347-9 (PMC12764432; doi:10.1038/s41590-025-02347-9)
Supplement: Supplementary file 2 — Reporting Summary [file 41590_2025_2347_MOESM2_ESM.pdf]

nature portfolio

Corresponding author(s): Laura K. Mackay
Last updated by author(s): Sep 22, 2024

Reporting Summary

Nature Portfolio wishes to improve the reproducibility of the work that we publish. This form provides structure for consistency and transparency in reporting. For further information on Nature Portfolio policies, see our Editorial Policies and the Editorial Policy Checklist.

Statistics

For all statistical analyses, confirm that the following items are present in the figure legend, table legend, main text, or Methods section.

n/a	Confirmed	
☐	☒	The exact sample size (*n*) for each experimental group/condition, given as a discrete number and unit of measurement
☐	☒	A statement on whether measurements were taken from distinct samples or whether the same sample was measured repeatedly
☐	☒	The statistical test(s) used AND whether they are one- or two-sided<br>*Only common tests should be described solely by name; describe more complex techniques in the Methods section.*
☒	☐	A description of all covariates tested
☐	☒	A description of any assumptions or corrections, such as tests of normality and adjustment for multiple comparisons
☐	☒	A full description of the statistical parameters including central tendency (e.g. means) or other basic estimates (e.g. regression coefficient) AND variation (e.g. standard deviation) or associated estimates of uncertainty (e.g. confidence intervals)
☐	☒	For null hypothesis testing, the test statistic (e.g. *F*, *t*, *r*) with confidence intervals, effect sizes, degrees of freedom and *P* value noted<br>*Give P values as exact values whenever suitable.*
☒	☐	For Bayesian analysis, information on the choice of priors and Markov chain Monte Carlo settings
☒	☐	For hierarchical and complex designs, identification of the appropriate level for tests and full reporting of outcomes
☒	☐	Estimates of effect sizes (e.g. Cohen's *d*, Pearson's *r*), indicating how they were calculated

Our web collection on statistics for biologists contains articles on many of the points above.

Software and code

Policy information about availability of computer code

Data collection	Flow cytometry data were collected with SpectroFlo v3.0 (Cytek). CITEseq data were collected using chromium controller (10X Genomics). Cyclic IF data was obtained using a Cytefinder II HT (Rarecyte) slide scanning fluorescent microscope.
Data analysis	Spectral flow cytometry data was unmixed with SpectroFlo v3.0 software, and then analysed with FlowJo (v.10.10.0; Treestar) or OMIQ. CITEseq data were analysed with CellRanger (6.1.2), Seurat (4.3.0), R(4.2), Harmony as well as other tools cited in the methods section. CycIF data were analysed using Cecelia. All code is available on Github.

For manuscripts utilizing custom algorithms or software that are central to the research but not yet described in published literature, software must be made available to editors and reviewers. We strongly encourage code deposition in a community repository (e.g. GitHub). See the Nature Portfolio guidelines for submitting code & software for further information.

Data

Policy information about availability of data

All manuscripts must include a data availability statement. This statement should provide the following information, where applicable:

- Accession codes, unique identifiers, or web links for publicly available datasets
- A description of any restrictions on data availability
- For clinical datasets or third party data, please ensure that the statement adheres to our policy

All data are available from the corresponding author upon reasonable request. The single-cell CITE and RNA-sequencing data generated from this study is available in GEO. Source data will be provided with this paper.

Research involving human participants, their data, or biological material

Policy information about studies with human participants or human data. See also policy information about sex, gender (identity/presentation), and sexual orientation and race, ethnicity and racism.

Reporting on sex and gender	Breast cancer patients were all female, except 1 male donor used for CycIF imaging.
Reporting on race, ethnicity, or other socially relevant groupings	Race and ethnicity were not recorded or relevant. Researchers were blinded to these characteristics.
Population characteristics	Medical diagnosis and treatments
Recruitment	Patients with breast cancer undergoing mastectomy or lumpectomy were recruited by Dr. Simon Tsao, at St Vincent's Hospital, Melbourne, Australia. Colorectal cancer patients with metastases to the liver undergoing surgical resection of tumours were recruited by Dr Marcos Perini without restriction or bias.
Ethics oversight	All study on human specimens was approved by the Human Research Ethics Committee of the University of Melbourne (ID nos. 13009 and 14517). All participating patients provided written informed consent.

Note that full information on the approval of the study protocol must also be provided in the manuscript.

Field-specific reporting

Please select the one below that is the best fit for your research. If you are not sure, read the appropriate sections before making your selection.

☒ Life sciences ☐ Behavioural & social sciences ☐ Ecological, evolutionary & environmental sciences

For a reference copy of the document with all sections, see nature.com/documents/nr-reporting-summary-flat.pdf

Life sciences study design

All studies must disclose on these points even when the disclosure is negative.

Sample size	Sample sizes are all reported in the respective figure legends and methods. For mouse experiments, sample sizes were chosen based on previous analyses in Prof. Mackay's laboratory demonstrating sufficient power to detect effects in similarly designed studies.<br>Human experiments used a minimum of 5 independent donors, and findings were repeated and validated across different tumour settings, and cross-validated in public datasets
Data exclusions	No data were excluded from the respective analyses
Replication	All replication has been stated in the respective figure legends and methods. All mouse experiments were repeated a minimum of two times.
Randomization	Mice were randomised to individual groups.
Blinding	Investigators were not blinded to groups, as this was not necessary or relevant for the study.

Reporting for specific materials, systems and methods

We require information from authors about some types of materials, experimental systems and methods used in many studies. Here, indicate whether each material, system or method listed is relevant to your study. If you are not sure if a list item applies to your research, read the appropriate section before selecting a response.

Materials & experimental systems

n/a	Involved in the study
☐	☒ Antibodies
☐	☒ Eukaryotic cell lines
☒	☐ Palaeontology and archaeology
☐	☒ Animals and other organisms
☒	☐ Clinical data
☒	☐ Dual use research of concern
☒	☐ Plants

Methods

n/a	Involved in the study
☒	☐ ChIP-seq
☐	☒ Flow cytometry
☒	☐ MRI-based neuroimaging

Antibodies

Antibodies used

Species Specificity Fluorochrome Clone Source Catalog # RRID Dilution
Human CD4 BUV496 SK3 BD 612936 AB_2870220 50
Human CD3 BUV737 UCHT1 BD 612750 AB_2870081 50
Human CD8b BUV805 2ST8.547 BD 749366 AB_2873737 200
Human CD103 BV480 Ber-ACT8 BD 746472 AB_2743774 25
Human CD45RA BV510 HI100 Biolegend 304138 AB_2561460 50
Human CCR7 PE-CF594 150503 BD 562381 AB_11153301 50
Human CD161 PE-Cy7 HP-3G10 Biolegend 339918 AB_11126745 50
Human TCR Va7.2 BV711 3C10 Biolegend 351732 AB_2629680 50
Human CD69 APC-Cy7 FN50 BD 557756 AB_396862 25
Human CD8a BV650 RPA-T8 Biolegend 301042 AB_2563505 100
Human CD73 BV421 AD2 Biolegend 344008 AB_11204424 25
Human CD38 ef450 HIT2 eBioscience 48-0389-42 AB_11151696 25
Human CD161 BV605 HP-3G10 Biolegend 339916 AB_2563607 25
Human CCR7 BV650 GO43H7 Biolegend 353234 AB_2563867 25
Human IFNg BV786 4S.B3 Biolegend 502509 AB_315234 25
Human TNF FITC MAb11 BD 562082 AB_395443 50
Human IL-2 PerCP-ef710 MQ1-17H12 eBioscience 46-7029-42 AB_1834419 25
Human CD101 PE BB27 Biolegend 331012 AB_2716107 25
Human CD39 PE-Dazzle594 A1 Biolegend 328224 AB_2564319 25
Human CD107a PE-Cy5 eBioH4A3 Invitrogen 15-1079-42 AB_10547280 100
Human IL-17A PE-Cy7 BL168 Biolegend 512315 AB_2295923 25
Human CD94 APC DX22 Biolegend 305508 AB_2133129 25
Mouse CD44 BUV395 IM7 BD 568507 AB_2739963 400
Mouse Va2 BUV615 B20.1 BD 751416 AB_2875415 400
Mouse CD4 BUV805 RM4-4 BD 741913 AB_2871227 400
Mouse CD103 BV480 M290 BD 566118 AB_2739520 200
Mouse CD39 BV711 Y23-1185 BD 567295 AB_2916538 400
Mouse CD8b BV750 H35-17.2 BD 747505 AB_2872172 500
Mouse PD-1 BV785 29F.1A13 Biolegend 135225 AB_2563680 200
Mouse TCF1 AF488 C63D9 Cell Signalling 6444S AB_2797627 200
Mouse CD90.2 PerCP 53-2.1 Biolegend 140316 AB_10642813 400
Mouse CD69 PE-Cy5 H1.2F3 Biolegend 104510 AB_313113 200
Mouse Tim3 PE-Cy7 RMT3-23 Biolegend 119716 AB_2571933 200
Mouse TOX e660 TXRX10 Thermo 50-6502-82 AB_2574265 200
Mouse CD45.1 APC-R700 A20 BD 565813 AB_2744397 200
Mouse CD45.2 PE-Cy7 104 Biolegend 109830 AB_1186098 200
Mouse CD39 PE 24DMS1 eBioscience 12-0391-82 AB_1210740 400
Mouse CD45.2 SparkNIR685 104 Biolegend 109864 AB_2876424 200
Mouse CD4 BUV496 RM4-5 BD 741050 AB_2870665 400
Mouse CD8b AF700 YTS156.7.7 Biolegend 126618 AB_2563949 400
Mouse CD45.1 PE-Cy7 A20 BD 560578 AB_1727488 200
Mouse CD39 AF700 24DMS1 eBioscience 56-0391-82 AB_2662998 200
Mouse CD103 PerCP-Cy5.5 2E7 Biolegend 121416 AB_2128621 200
Mouse Va2 BV421 B20.1 BD 562944 AB_2737910 400
Mouse CD49a BUV395 Ha31/8 BD 740262 AB_2740005 200
Mouse Ly108 BV421 13G3 BD 740090 AB_2739850 200
Mouse CXCR6 PE-ef610 SA051D1 Biolegend 151104 AB_2566546 200
Mouse TCRb APC-Fire750 H57-597 BD 561080 AB_398534 200
Mouse CD45.1 APC A20 Biolegend 110714 AB_313503 200
Mouse CD69 PE-Cy7 H1.2F3 BD 552879 AB_394508 200
Mouse CD103 FITC 2E7 Thermo 11-1031-82 AB_465176 200
Streptavidin PE BD 554061 AB_10053328
SIINFEKL monomer NIH Tetramer core 400
Human CD39 AB_2889212 none EPR20627 Abcam ab223842
Human Rabbit IgG AB_2534114 Alexa Fluor 488 polyclonal Invitrogen A-11070
Human CD105 AB_354598 none polyclonal R&D Systems AF1097
Human Goat IgG AB_2535853 Alexa Fluor 555 polyclonal Thermo Fisher Scientific A-21432

Human CD33 AB_2943218 none PWS44 BioCare ACI 3116 A
Human Mouse IgG AB_2535806 Alexa Fluor 647 polyclonal Thermo Fisher Scientific A-21237
Human CD45RO AB_2237910 none UCHL1 Dako M0742
Human Mouse IgG2a AB_2338855 Alexa Fluor 488 polyclonal Jackson ImmunoResearch, Inc. 115-545-206
Human CD3E AB_3668891 Alexa Fluor 555 D7A6E Cell Signaling Technology 57869S
Human CD25 AB_2572851 none GLLZDMY Thermo Fisher Scientific 14-0256-82
Human Mouse IgG1 AB_2338916 Alexa Fluor 647 polyclonal Jackson ImmunoResearch, Inc. 115-605-205
Human phospho-Histone 3 AB_3668896 Alexa Fluor 750 D2C8 Cell Signaling Technology 43185S
Human NT5E/CD73 AB_2716625 Alexa Fluor 488 D7F9A Cell Signaling Technology 14627BC
Human Ki-67 AB_2797836 phycoerythrin D3B5 Cell Signaling Technology 12160S
Human CD31 AB_2857973 Alexa Fluor 647 EPR3094 Abcam ab218582
Human Cytokeratin (pan) AB_2868569 Alexa Fluor 750 AE-1/AE-3 Novus Biologicals NBP2-33200AF750
Human CD8a AB_2574412 Alexa Fluor 488 AMC908 Thermo Fisher Scientific 53-0008-80
Human GZMB Alexa Fluor 555 D6E9W Cell Signaling Technology 29268S
Human CD103 AB_2884945 Alexa Fluor 647 EPR4166(2) Abcam ab225153
Human LAG3 Alexa Fluor 488 EPR4392(2) Abcam ab225277
Human CD11c AB_3331656 Alexa Fluor 555 D3V1E Cell Signaling Technology 77882S
Human PD-1 AB_2728811 Alexa Fluor 647 EPR4877(2) Abcam ab201825
Human Alpha-actin-2 AB_2868436 Alexa Fluor 750 1A4 R&D Systems IC1420S-025
Human TIM3 AB_2799468 Alexa Fluor 488 D5D5R Cell Signaling Technology 54669S
Human Catenin beta-1 AB_2868511 Alexa Fluor 555 E247 Abcam ab202496
Human CD69 Alexa Fluor 647 EPR21814 Abcam ab313397
Human TCF1/TCF7 AB_2797627 Alexa Fluor 488 C63D9 Cell Signaling Technology 6444S
Human FOXP3 AB_2573609 eFluor 570 236A/E7 eBioscience 41-4777-82
Human GNLY Alexa Fluor 647 E2T3D Cell Signaling Technology 64458S
Human CD68 AB_2798886 Alexa Fluor 488 D4B9C Cell Signaling Technology 24850S
Human CD66b AB_2750201 phycoerythrin 6/40c BioLegend 392903
Human CD20 AB_11151691 eFluor 660 L26 eBioscience 50-0202-82
Human Mast Cell Tryptase AB_2943323 Alexa Fluor 790 AA1 Santa Cruz Biotechnology sc-59587 AF790
Human Vimentin AB_10829352 Alexa Fluor 488 D21H3 Cell Signaling Technology 9854S
Human HLA -DRA AB_2889281 Alexa Fluor 555 EPR3692 Abcam ab215312
Human HER2 AB_2889201 Alexa Fluor 647 EPR19547-12 Abcam ab225510
Human CD4 AB_2728839 Alexa Fluor 488 R&D Systems FAB8165G
Human CD206 Alexa Fluor 555 E2L9N Cell Signaling Technology 48352S
Human Progesterone receptor AB_2890175 Alexa Fluor 647 YR85 Abcam ab199455
Human c-Jun (pS73) AB_2798004 Alexa Fluor 488 D47G9 Cell Signaling Technology 12714S
Human phospho-MAPK Alexa Fluor 555 D13.14.4E Cell Signaling Technology 76032S
Human YAP1 AB_2728837 Alexa Fluor 647 D8H1X Cell Signaling Technology 38707S
Human PCNA AB_3669072 Alexa Fluor 750 PC10 Cell Signaling Technology 24114BC
Human HLA-A and HLA-B AB_2943099 Alexa Fluor 488 EPR1394Y Abcam ab198376
Human IBA1 AB_2943227 Alexa Fluor 555 E4O4W Cell Signaling Technology 36618S
Human ER alpha AB_2728817 Alexa Fluor 647 EPR4097 Abcam ab205851
Human CD15 AB_493257 Alexa Fluor 488 HI98 BioLegend 301910
Human CD7 AB_2889193 Alexa Fluor 647 EPR4242 Abcam ab199023
Human KLF2 Alexa Fluor 555 E7K8Y Cell Signaling Technology 73198BC
Human CD11b AB_2637195 Alexa Fluor 488 C67F154 Thermo Fisher Scientific (eBioscience) 53-0196-82
Human NKG2A AB_2943211 Alexa Fluor 647 EPR23737-127 Abcam ab300745
Human CD94 Alexa Fluor 555 EPR21003 Abcam ab318870
Human MX1 AB_2799122 Alexa Fluor 488 D3W7I Cell Signaling Technology 79373BC
Human ICOS AB_2800142 Alexa Fluor 555 D1K2T Cell Signaling Technology 79403BC
Human CD16 AB_626925 Alexa Fluor 647 DJ130c Santa Cruz Biotechnology sc-20052 AF647

Validation

Antibodies used for flow cytometry were validated for the corresponding application by the manufacturers. Antibodies were also tested internally for the corresponding applications internally through preliminary experiments using primary cells from humans and mice before performing experiments using patient cells.

Eukaryotic cell lines

Policy information about cell lines and Sex and Gender in Research

Cell line source(s)

AT3-OVA cells were provided by Professor Philip Darcy, Peter MacCallum Cancer Centre. B16F1-gB.GFP (B16-gB) cells were provided by Jason Waithman (University of Western Australia).

Authentication

These cell lines were not authenticated.

Mycoplasma contamination

These cell lines were tested negative for mycoplasma contamination before experimental use.

Commonly misidentified lines
(See ICLAC register)

No commonly misidentified cell lines were used

Animals and other research organisms

Policy information about studies involving animals; ARRIVE guidelines recommended for reporting animal research, and Sex and Gender in Research

Laboratory animals	C57Bl/6 female mice were used between 7-12 weeks of age at the beginning of the experiments
Wild animals	NA
Reporting on sex	All mice used were females due to the focus on breast cancer models.
Field-collected samples	NA
Ethics oversight	All animal experiments were approved by The University of Melbourne Animal Ethics Committee (ID nos. 21651 and 21938).

Note that full information on the approval of the study protocol must also be provided in the manuscript.

Plants

Seed stocks	*Report on the source of all seed stocks or other plant material used. If applicable, state the seed stock centre and catalogue number. If plant specimens were collected from the field, describe the collection location, date and sampling procedures.*
Novel plant genotypes	*Describe the methods by which all novel plant genotypes were produced. This includes those generated by transgenic approaches, gene editing, chemical/radiation-based mutagenesis and hybridization. For transgenic lines, describe the transformation method, the number of independent lines analyzed and the generation upon which experiments were performed. For gene-edited lines, describe the editor used, the endogenous sequence targeted for editing, the targeting guide RNA sequence (if applicable) and how the editor was applied.*
Authentication	*Describe any authentication procedures for each seed stock used or novel genotype generated. Describe any experiments used to assess the effect of a mutation and, where applicable, how potential secondary effects (e.g. second site T-DNA insertions, mosiacism, off-target gene editing) were examined.*

Flow Cytometry

Plots

Confirm that:

☒ The axis labels state the marker and fluorochrome used (e.g. CD4-FITC).

☒ The axis scales are clearly visible. Include numbers along axes only for bottom left plot of group (a 'group' is an analysis of identical markers).

☒ All plots are contour plots with outliers or pseudocolor plots.

☒ A numerical value for number of cells or percentage (with statistics) is provided.

Methodology

Sample preparation	Cells were isolated from tissues as described in the methods. Single cell suspensions were then stained with surface stain and fixable Live/Dead stain (Zombie Near IR, Zombie Aqua, Zombie Yellow). When needed, cells were fixed/permeabilised with FoxP3 transcription factor kit (eBioscience) and stained with intracellular antibodies.
Instrument	Cytek Aurora Flow Cytometer
Software	SpectroFlo v3.0 software was used for spectral unmixing, and then analysed with FlowJo (v.10.10.0; Treestar) or OMIQ
Cell population abundance	Subset abundance was determined by manual gating in FlowJo or high-dimensional analysis using OMIQ, with clustering using the FlowSOM algorithm
Gating strategy	Lymphocytes were gating based on FSC/SSC, singlets gated based on FSC-W vs FSC-H and SSC-W vs SSC-H. Live cells were gated based on live cell exclusion. Further gates are described for respective figures

☒ Tick this box to confirm that a figure exemplifying the gating strategy is provided in the Supplementary Information.
